# Immune and inflammatory mechanisms of abdominal aortic aneurysm

**DOI:** 10.3389/fimmu.2022.989933

**Published:** 2022-10-05

**Authors:** Ana Cristina Márquez-Sánchez, Ekaterina K. Koltsova

**Affiliations:** Department of Medicine, Department of Biomedical Sciences, Research Division in Immunology, Cedars-Sinai Cancer Institute, Smidt Heart Institute, Cedars-Sinai Medical Center, Los Angeles, CA, United States

**Keywords:** abdominal aortic aneurysm, inflammation, immune cells, cytokines, microbiota, vascular immunology, tissue microenvironment

## Abstract

Abdominal aortic aneurysm (AAA) is a life-threatening cardiovascular disease. Immune-mediated infiltration and a destruction of the aortic wall during AAA development plays significant role in the pathogenesis of this disease. While various immune cells had been found in AAA, the mechanisms of their activation and function are still far from being understood. A better understanding of mechanisms regulating the development of aberrant immune cell activation in AAA is essential for the development of novel preventive and therapeutic approaches. In this review we summarize current knowledge about the role of immune cells in AAA and discuss how pathogenic immune cell activation is regulated in this disease.

## Introduction

Cardiovascular diseases (CVD) are the leading cause of death globally with an estimated ~18 millions of annual deaths (up to 32% of global deaths) (1) and high prevalence in both high and low income countries (2). Abdominal aortic aneurysm (AAA) is a CVD characterized by abdominal aorta dilatation exceeding the diameter of aorta by 50%, caused by immune cell-mediated inflammation and degradation of the medial layer; eventually followed by aortic rupture and bleeding that is often sudden and fatal. AAA affects about 5% of the population and represent 15th most frequent cause of mortality in the US, where each year ~200,000 people are diagnosed with AAA. Smoking, age (> 60 years old), hypertension, atherosclerosis, and male gender are established AAA risk factors (3–8). Although new potential therapies have been recently proposed for AAA treatment, including nanoparticles loaded with antihypertensive drugs, statins or inhibitors of vascular endothelial growth factor receptor (VEGFR) (9, 10), the current standard of care is still mostly limited to surgery at late stages of the disease (11, 12). Despite significant progress in the understanding of pathophysiology of AAA (3, 13–16), immune and inflammatory mechanisms controlling this disease pathogenesis only recently started to come to light as a mainstream and pivotal players. Nowadays, chronic inflammation caused by the infiltration and activation of various immune cells is an important driver of AAA (3, 5, 6). Yet, factors regulating immune cell recruitment and activation in AAA remains incompletely understood. Here we discuss recent data on immune and inflammatory mechanisms implicated to the control of AAA development and briefly highlight local and systemic factors impacting immune cell activation in this disease.

## Immune cells in abdominal aortic aneurysm

### Innate immune cells

**Figure 1:**
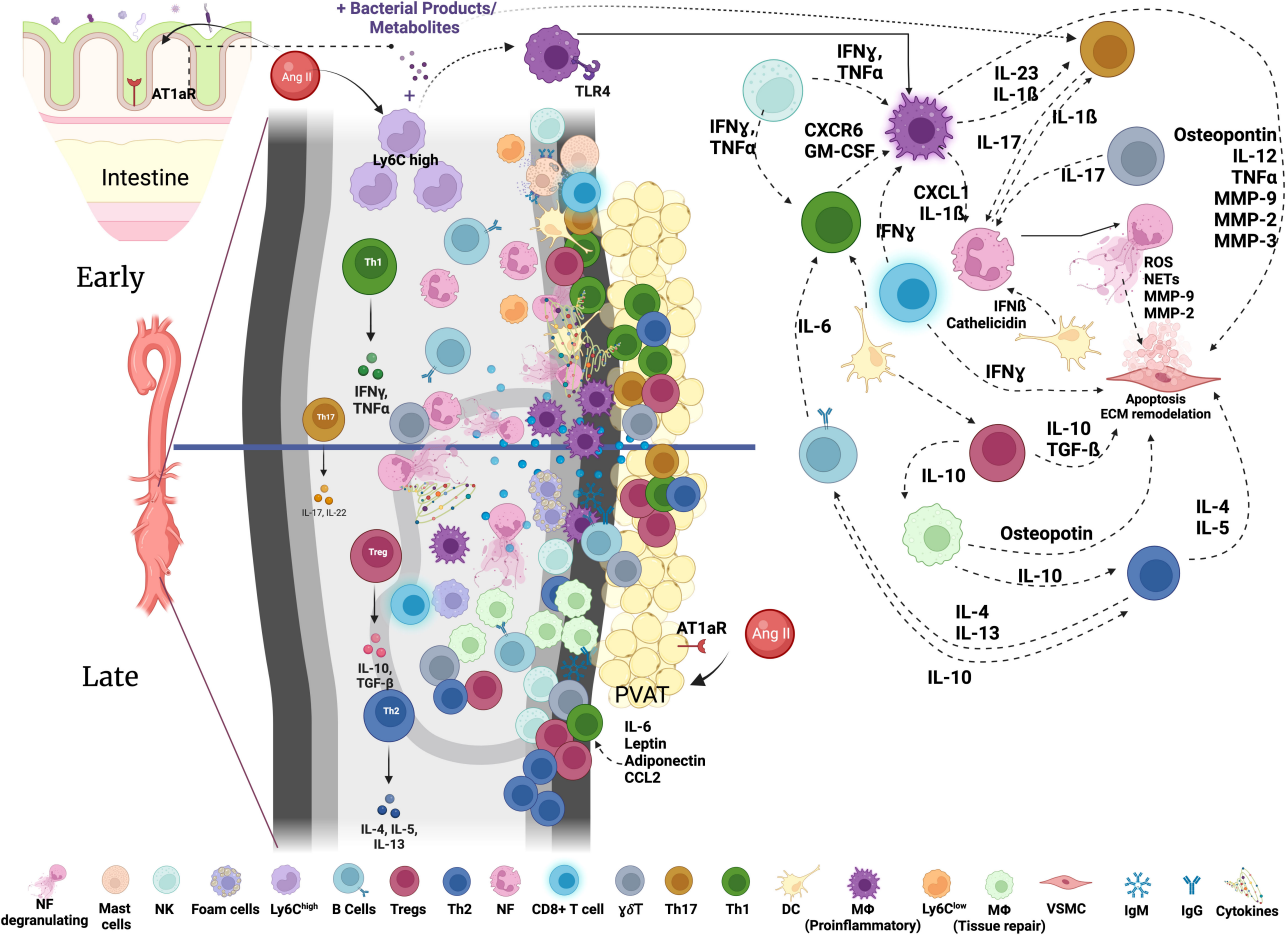
Immune networks in aortic abdominal aneurysm. Various immune cells are found in the aorta with AAA. The composition and activation status of immune cells infiltrating the aortic wall during AAA development is dynamic and changes through the course of disease development. Activated immune cells contribute to the inflammatory environment in the aortic wall and VSMC apoptosis resulting in the destruction of the aorta and progressive growth of AAA eventually leading to rupture. NK, Natural Killer; MF, macrophages; Tregs, T regulatory cells; NF, neutrophils; γδ T cells, Th17, T helper 17 cells; Th1, T helper 1 cells; Th2, T helper 2 cells; DC, dendritic cells; VSMC, vascular smooth muscle cells; IgM, immunoglobulin M; IgG, immunoglobulin G; MMPs, matrix metalloproteinases; GM-CSF, granulocyte-macrophage colony-stimulating factor; IL, Interleukin; ECM, Extracellular Matrix; AT1aR, Angiotensin II Receptor Type 1; TLR, Toll-like Receptor; PVAT, perivascular adipose tissue; Ang II, Angiotensin II.

Myeloid cells, including neutrophils, monocytes, macrophages and dendritic cells (DC) play diverse and important roles in inflammation, immunity and tissue repair (17). They also contribute to the aortic inflammation and vessel destruction during AAA (6, 16, 18, 19). Early myeloid cell infiltration in the aortic wall is considered to be a hallmark of AAA development both in mice and humans (20, 21), suggesting that these cells could contribute to initial steps of aortic wall destruction (**Figure 1**).

#### Neutrophils

Neutrophils, cells of bone marrow origin, are the most abundant circulating leukocytes in the human immune system and the first effector cells to be recruited to the site of injury, infection or inflammation. Neutrophils represent one of the most prevalent cell populations found in the aneurysm and are detected even in early lesions (19). Neutrophils are capable to release different types of granules containing various bioactive molecules such as myeloperoxidase (MPO), neutrophil elastase (NE), defensins, cathepsin G, azurodicin, and endotoxin-neutralizing proteins (22), NADPH oxidase (NOX) and matrix metalloproteinases (MMPs) (23–25). The latter are highly abundant in human and mouse AAA tissues (26–28). Activated neutrophils produce extracellular traps (NETs), a web-like defense structures to trap foreign cells, growth factors, cytokines, proteases and expose them to effector protein (29). NETs are long intersecting fibers consisting of released neutrophil DNA, and histones 3 and 4 (H3 and H4) as well as cytoplasm-derived effector molecules MPO, NE, and cathepsin G (29). Neutrophils play essential and diverse roles in CVD (5, 18, 30–32). NETs production in atherosclerosis is triggered by inflammatory stimuli including LPS and cholesterol crystals (29, 31, 33, 34). Hypochlorous acid generated by MPO oxidizes circulating LDL, contributing to the activation of macrophages and foam cell formation (22). Neutrophil NETs promote inflammation facilitating the activation of Th17 cells and macrophages regulating the release of IL-1β, IL-18 and other pro-inflammatory cytokines (29). Moreover, NETs induce apoptosis of vascular smooth muscle cells (VSMC) leading to the thinning of the fibrous cap and eventual plaque rupture (29). In myocardial infarction (MI), the recruitment and infiltration of neutrophils has been also associated with cardiac damage, but nevertheless neutrophils are needed for healing processes after MI. Neutropenic mice were characterized by increased fibrosis and heart failure because of altered macrophage polarization skewed to a highly inflammatory state with low phagocytic capacity (29).

NLR (Neutrophil to Lymphocyte Ratio) was recently suggested as a prognostic marker for AAA patients, where high frequency of neutrophils in circulation in comparison with lymphocytes predicted a poor prognosis and mortality in patients with ruptured aneurysm (35–38). It has been shown that neutrophils, neutrophil-derived IL-8 and NETs elements were elevated in plasma and tissue of patients with AAA suggesting enhanced neutrophil activation (18, 33, 34). The recruitment of neutrophils is facilitated by CXCL1, CCL2, CCL5 and CXCL8 chemokines (22, 39) that are mainly produced by pro-inflammatory macrophages and are elevated in serum and aorta from humans (40–42) and mice with AAA (43). The depletion of neutrophils or genetic ablation of neutrophil-specific genes (e.g., MPO, MMP-9) attenuates AAA development, suggesting overall pathogenic role for these cells in AAA (3, 19, 44, 45). Several types of neutrophil-derived effector molecules including NETs were detected in the intraluminal thrombus of patients with advanced AAA (23, 31). In elastase mouse model, NETs citrullinated (cit-) H3 and neutrophil elastase were found in the adventitia and at the border of intima and media; and depositions of cit- H3 and H4 were found in the intraluminal thrombus where they co-localized with IL-1β (18). Both Angiotensin (Ang) II and NADPH oxidase-derived ROS were also shown to stimulate NETs formation (38) and inhibition of NETosis by altering the function of PAD enzyme family significantly limits aneurysm development (46, 47). Altogether, these studies imply neutrophils as important inflammatory regulators of AAA. Nevertheless, mechanisms triggering NETosis as well as interaction of neutrophils with other cell types within AAA lesions require further investigation. It also remains to be determined whether neutrophils initiate the destruction of the aortic wall in AAA or simply work as a first responders to the injury driven by some other factors.

#### Monocytes

Monocytes, originated from the bone marrow, play crucial roles in host defense and contribute to various chronic inflammatory diseases, including CVD (6, 48–60). In humans, three populations of monocytes have been described based on CD14 and CD16 surface expression. Classical monocytes represent up to 90% of circulating monocytes and are characterized by CD14^++^ and CD16^-^ expression, high surface expression of CCR2, CD62L (L-selectin), and low levels of CX3CR1. Non-classical monocytes are characterized by CD14^+^ CD16^++^ surface phenotype, high levels of CX3CR1 and low CCR2. The third population has an “intermediate” phenotype of CD14^++^ CD16^+^ and was suggested to have pro-inflammatory and enhanced phagocytic properties (6). In mice, two homologous populations Ly6C^high^ (classical) and Ly6C^low^ (non-classical) had been described. Ly6C^high^ monocytes are equivalent to human classical monocytes, and were shown to promote inflammatory responses and perform antimicrobial and phagocytic functions. Ly6C^low^ monocytes, corresponding to human non-classical monocytes, are involved in vessel patrolling, immune surveillance and tissue repair. Monocytes had been implicated to the pathogenesis of various CVD, and elevated numbers of circulating monocytes had been associated with atherosclerosis, myocardial infarction and AAA (6, 20). Several studies described changes in circulating monocytes in patients with AAA. While one study showed a reduction of classical monocytes in circulation and augmented proportion of intermediate monocytes, an increase of classical monocytes or no alterations in their presence had been also reported (16, 61–63). In mice Ang II infusion, which heightens blood pressure and drives AAA development, was shown to increase number of circulating Ly6C^high^ monocytes (6, 16), while the administration of angiopoietin 2 reduced circulating Ly6C^high^ monocytes and attenuated AAA (64). Moreover, mice lacking CCR2 were protected from AAA due to the limited recruitment of monocytes to the aorta along with low IL-6 and CCL2 expression (65). The role of CD11b, an integrin subunit expressed on monocytes, but also on macrophages and facilitating the recruitment of immune cells to the site of inflammation, had been investigated in AAA. Higher levels of CD11b on circulating monocytes from patients with AAA compared to healthy subjects have been reported (6, 26). *In vitro* experiments showed that monocytes from patients with AAA are more capable for adhesion and transmigration. However, the knockout of CD11b (*Itgam^-/-^*) did not significantly affect the incidence of AAA, but nevertheless reduced maximum abdominal aortic diameter, macrophage infiltration, MMP-9 and IL-6 expression, as well as elastin and collagen degradation (66).

Monocytes differentiate from hematopoietic stem and progenitor cells (HSPC); and Ang II was shown to activate those cells and stimulate myelopoiesis in the bone marrow (20). Another source of monocytes is a spleen where extramedullar hematopoiesis occurs (67, 68). Spleen-derived monocytes were shown to contribute to atherosclerosis, myocardial infarction and AAA (16, 69–71). Recent study demonstrated that in AAA acute mobilization of monocytes from the spleen to the circulation was dependent on Triggering Receptor Expressed on Myeloid Cells (TREM)1 and driven by Ang II *via* AT1R (21). Moreover, TREM 1 was also shown to regulate CD62L expression thereby facilitating monocyte infiltration into the aortic wall during AAA development (21).

The role of non-classical Ly6C^low^ monocytes in AAA had been also suggested in studies utilizing NR4A1 (Nuclear receptor subfamily 4 group A transcription factor) deficient mice. The reduction of Ly6C^low^ monocytes in these animals was associated with augmented AAA and elevated elastin destruction, suggesting potentially protective role of this monocyte subset (72). While these data imply that circulating monocytes play important roles in AAA, a detailed contribution of monocyte subsets, their cooperation with neutrophils and mechanisms controlling monocyte output in AAA remains to be elucidated.

#### Macrophages

Monocytes recruited to the aortic tissue are capable to further differentiate into macrophages or dendritic cells (DC) (73). Monocytes-derived macrophages are generally classified into “inflammatory” and “tissue repair” subsets, both of which had been implicated to AAA development (3, 6). While the localization of macrophages in AAA had been established (61, 74), macrophage polarization at different stages of the disease requires further investigation. Single-cell RNA sequencing of aortas showed about 5-fold early expansion of pro-inflammatory macrophages in CaCl_2_-induced AAA, while tissue-repair subset was not affected, suggesting the predominance of inflammatory macrophages (75).

*Inflammatory macrophages* acquire their phenotype upon activation with a vast variety of stimuli, including LPS, ROS, fatty acids, inflammatory cytokines or local hypoxia in the aortic wall (76–79). Inflammatory macrophages are characterized by elevated expression of pro-inflammatory mediators such as IL-1β, IL-6, TNF, IL-12, IL-23, MMPs, NOS2 and chemokines including CCL2 and CXCL1, in turn regulating the recruitment and activation of other immune cells as well as VSMC apoptosis (6). The expression of these pro-inflammatory molecules is particularly prominent at the advanced stages of AAA. Moreover, Ang II was suggested to promote macrophage activation *via* upregulation of TLR4 (80).

The role of various macrophage-derived cytokines and bioactive molecules had been investigated in multiple studies using pharmacological or genetic approaches, however many studies reported the conflicting results depending on the model used. For example, pharmacological blockade or knockout of IL-1β was shown to reduce AAA in CaCl_2_ model (81). However, recent study by Batra et al. using the same mouse model of AAA came to the opposite conclusions and showed that *Il1*β*^-/-^ or Il1r^-/-^* mice were not protected from the disease development, and *Il1r^-/-^* mice develop even larger AAA (82). Serum IL-1β levels were elevated in patients with AAA, which particularly was linked to rs35829419 polymorphism of NLRP3 common allele (83). Indeed heightened expression of NLRP3 inflammasome had been detected in AAA tissue (83). The genetic inactivation of NLRP3, or other inflammasome components (caspase-1 or ASC) reduced the incidence of AAA and ECM degradation in mice infused with Ang II (83). Activation of TLR4 can induce MMP9 expression in VSMC and macrophages, while expression of these entities was reversed in *Tlr4^-/-^* mice (80, 84). Similar results were observed with TLR4 antagonist, Eritoran (80). Recent study also documented higher TLR4 and MMP9 expression in lymphocytes rather than macrophages in human AAA (85).

Extracellular matrix degradation mediated by MMPs is a hallmark of AAA. Elevated serum MMP9 served as a prognostic marker for AAA (34), and it is known that genetic ablation of MMP9 and MMP2 halts AAA development in CaCl_2_ model (86, 87). Adoptively transferred WT macrophages promoted AAA growth in *Mmp9^-/-^* but not *Mmp2^-/-^* mice, suggesting the importance of MMPs in macrophages and collaborative action between MMP2 and MMP9 (86, 87).

TNF, a major macrophage-derived cytokine (88) was suggested to contribute to AAA in calcium chloride model (82), and its genetic ablation or pharmacological inhibition of TNF limited AAA development (89). At the same time the ablation of its main receptor TNFR1 (p55) in *Ldlr^-/-^* mice subjected to Ang II infusion did not significantly affect AAA formation, but strongly reduced atherosclerosis (90).

Elevated levels of IL-6, which is presumably myeloid cell derived, had been detected in serum and aortic tissue from patients with AAA (91). The production of IL-6 in aneurysm tissue is directly regulated by Ang II signaling (92); and IL-6 ablation protects from endothelial dysfunction induced by Ang II (93).

The role of IL-12 and IL-23 cytokines in AAA was suggested but different studies reported conflicting results. Antibody-mediated blockade of IL-12p40 at early stages of the AAA reduced aortic diameter and limited macrophage infiltration in elastase perfusion model (75). However, knockout of IL-12p40 resulted in augmented AAA development in Ang II model (94). While the observed difference in phenotypes may be due to different models used, it is important to note that p40 is a shared subunit between IL-23 and IL-12, and therefore genetic inactivation likely affects both cytokines. These data suggest that the results using neutralization or genetic knockout of one of the subunits of heterodimeric cytokines should be interpreted with caution. Moreover, both IL-12 and IL-23 are implicated in the regulation of microbiota, the effect of which has to be considered.

Recent studies identified among CD11b^+^CD68^+^Adgre1^+^ macrophages a unique subset marked by Netrin 1 expression. Netrin 1 (Ntn1) is a protein of the laminin family, which was suggested to be involved into the axon guidance and cell migration (95). *Ntn1*-positive macrophages expressed high amounts of pro-inflammatory and pro-angiogenic markers including MMP3, while macrophages with lower levels of *Ntn1* exhibited anti-inflammatory phenotype and expressed high level of macrophage mannose receptor 1 (*Mrc1*) and *Scd1, Cd36, Cydec, Dgat2, Apoc1* genes. Hematopoietic cell-specific Netrin-1 deficiency, meanwhile, prevented AAA formation (95).

Exosomes are lipid bilayer nanoparticles containing RNA and proteins that mediate cell-cell communication. They are produced by macrophages and other cell types as communication tools (96). Increased presence of exosomes has been associated with CVD, including AAA where exosomes were detected in the adventitia, mostly in areas of macrophage accumulation (97). *In vitro* experiments suggest that macrophage exosomes mediate VSMC migration and metabolism by modulating the expression of MMP2 in JNK- and p38-dependent manner. Inhibition of exosome formation by GW4869 reduced AAA progression, preserved elastin integrity and decreased MMP2 expression in a mouse model (96).

*Tissue repair macrophages*, known to perform tissue surveillance and tissue repair functions, are also implicated in AAA development (98). This subset of macrophages becomes more abundant at the late stages of the disease development, which might represent a compensatory mechanism to prevent further AAA expansion or rectify tissue injury. While Ang II stimulates Ly6C^high^ monocyte infiltration, it was also suggested to regulate the switch from pro-inflammatory to tissue repair macrophage phenotype (99). Also, coagulation factor XIIIa was shown to promote macrophage differentiation toward tissue repair phenotype in the aneurysm (6). Cytokines produced by this subset of macrophages, such as IL-10 and TGFβ, had been shown to play an important protective role in AAA. Increased IL-10 systemic level correlated with reduced AAA diameter and dissection in elastase model in rabbits (100) and *Apoe^-/-^* mice infused with Ang II (101). Infusion of recombinant IL-10 promoted smooth muscle cells proliferation in the aorta (100), and systemic induction of IL-10 by its overexpression increased accumulation of FoxP3^+^ Tregs in aortic tissue reducing the inflammation and diameter of AAA (101). Transforming growth factor (TGF-β) was shown play a protective role in AAA, since antibody neutralization of TGF-β augments AAA severity accompanied by macrophage accumulation in the aortic wall and enhanced ECM degradation (102). VSMC specific deletion of TGFβR2, however, seems to protect from the development of thoracic but not abdominal aneurysms, implying that TGFβ could act through different cell types at different part of the aorta (103).

*Tissue resident macrophages*. Aortas also harbor tissue resident macrophages, which originate from yolk sac during development (104, 105). These macrophages are also heterogeneous and can be polarized toward anti-inflammatory or tissue repair subsets. In cardiac repair, they play an important role in tissue regeneration and were shown to remove debris, regulate extracellular matrix (ECM), and stimulate cardiomyocytes proliferation (106), but their exact role in AAA has not been fully dissected yet. Single cell RNAseq analysis of elastase-driven AAA and healthy vessels revealed that CX3CR1^+^ (yolk-sac derived) macrophages are the most abundant subset in healthy aorta representing 62.5% of total macrophage population, while bone marrow derived macrophages (CCR2^+^Ly6C2^low^F4/80^low^CD11b^low^H2-Aa^low^) start to dominate in AAA lesions (107). Another tissue resident subset of Flt3^+^ macrophages is increased in AAA and expresses pro- and anti-inflammatory cytokines such as CCL3, IL-1β and IL-10 (107), suggesting their contribution to cell recruitment and activation. A trans-differentiation of VSMCs toward a “macrophage-like” phenotype was demonstrated in atherosclerotic disease (108), however the relevance of this mechanism to AAA remains to be determined.

The interplay between monocytes, macrophages and neutrophils could also be implicated to their reciprocal activation during AAA pathogenesis. Early monocytes infiltration in the aortic wall in AAA and differentiation toward inflammatory macrophage subset with the subsequent production of CXCL1 may further facilitate neutrophil recruitment, contributing to the aortic wall destruction. Conversely, neutrophils produce IL-6 known to contribute to pro-inflammatory macrophage activation (6). Moreover, macrophage macropinocytosis was linked to the engulfment of NETs, and a negative correlation between the density of macrophages and NETs in AAA was observed (109).

#### Dendritic cells

Dendritic cells (DC) are professional antigen presenting cells that link innate and adaptive immune responses (6). DC activate T cells and also contribute to innate immune responses *via* secretion of pro-inflammatory cytokines, including TNF, IL-12, IL-23 and others as well as chemokines (105, 110). Dendritic cells can be divided on conventional DC, plasmacytoid DC, lymphoid DC and inflammatory DC subsets. The latter differentiate from the recruited monocytes at the site of inflammation (111). DC had been detected in AAA (45, 112) and depletion of CD11c^+^ DC using DTR-driven approaches led to the reduction in maximum diameter of AAA in Ang II-driven model (112). Depletion of DC lowered numbers of circulating CD44^high^ CD62L^low^ effector CD4 T cells, CD44^high^ CD62L^low^ effector CD8 T cells and B cells. Moreover, DC depletion also attenuated SRA matrix degradation by limiting neutrophil elastase activity, resulting in limited elastin degradation and heightened collagen content (112). Plasmacytoid DC activation in AAA has been linked to NET formation due to their ability to produce cathelicidin and type I IFNs (45). Therefore, DC were suggested to promote lymphocyte and neutrophil infiltration and activation, and regulate matrix content and organization. Nevertheless, the putative self-antigens presented by DC in AAA are not known and mechanisms driving initial DC accumulation and activation in AAA remains to be elucidated.

#### Mast cells

Mast cells had been detected in AAA lesions in outer media and adventitia, and their number correlated with AAA diameter (113). Mast cells are known to produce proteases such as tryptase and chymase, inhibition of which is explored as a therapeutic approach for AAA in animal models (114). Immunoglobulin E (IgE) is a signature molecule of allergic responses activating FcϵR1 on mast cells. *Apoe^-/-^Ige^-/-^* mice infused with Ang-II or treated with CaCl_2_ were protected from AAA and neutralization of IgE by antibodies reduced AAA formation and inflammation in the aorta (115, 116). Amelioration of the disease was accompanied by limited recruitment of neutrophils and lowered expression of MIP-2a and CXCL5 in AAA tissue (116). One of the suggested mechanisms was *via* TNF produced by mast cells, which was regulated by metalloendopeptidase Meprin-α (Mep1A). Mast cell-derived TNF regulated MMP2 production and VSMC apoptosis in AAA; and the Mep1A deficiency ameliorated the disease (117). These observations provide an important largely unexplored link between allergic inflammation and AAA development and warrant detailed investigation in future studies.

#### NK and ILC cells

Both Natural killer (NK) and Innate lymphoid cells (ILC) are professional innate cytotoxic cells capable of producing cytokines, such as IFNγ, or cytotoxic molecules, such as FasL (CD95L), perforin and granzymes. They typically act to eliminate infected, stressed, senescent or transformed cells (118). NK cells represent a potent source of inflammatory IFNγ, and their pathologic role in atherosclerosis had been previously suggested (119). Immunohistochemistry and microarray analysis of human AAA tissue revealed elevated presence of NK cells in AAA tissue along with upregulated granzyme B and other cytotoxic markers (120, 121). Hematopoietic deficiency of CD95L, a transmembrane protein regulating cell death or pro-survival pathways (122), significantly reduced AAA formation in CaCl_2_ model, which was associated with lowered infiltration of macrophages and T cells along with limited MMP-2 and MMP-9 expression (123).

Innate lymphoid cells (ILC) comprise of three major populations (ILC1, ILC2 and ILC3) which are characterized by distinct functions and spectrum of produced cytokines (124). ILCs can be typically found at mucosal surfaces, in the adventitia of arteries, pericardium, adipose tissue as well as liver (124, 125). While ILC1 are known producers of IFNγ, ILC2 represents a critical source of type 2 cytokines such as IL-4, IL-5, IL-9 and IL-13 (125). ILC2 were implicated to the regulation of metabolic homeostasis, obesity, helminth infection and allergic lung inflammation (126–128). In atherosclerosis-prone mice fed with high fat diet (HFD) ILC2 cells were found in para-aortic fat tissue and were characterized by pro-inflammatory gene expression profile (124). Also, NK cells expressing IL-4, IL-5 and IL-13 were associated with the development of AAA in early studies, nowadays would be probably classified as ILC2 (120). NK cell mediated IL-13 production can induce MMP-2, -9, -13 and -14 in pulmonary diseases (129), thereby hinting at its potential role in AAA progression *via* similar mechanisms. ILC3 are RORγt-dependent cells, which produce IL-17A and IL-22 cytokines (130). The role of these cells in CVD only recently attracted attention and was discussed elsewhere (131), while their role in AAA have not yet been examined.

#### iNKT

Invariant Natural Killer T (iNKT) cells express TCRβ and NK1.1 surface markers. NKT cells recognize non-classical antigens, including lipids, presented in the context of MHC-I and MHC-I-like molecules, including CD1d (132). In vascular diseases, NKT cells has been implicated in the progression of atherosclerosis (57, 133). In human AAA tissue, an increased proportion of activated Vα24Jα18^+^NKT subsets in the media was reported (134). Elevated presence of iNKT cells in AAA had been also found in *Apoe^-/-^* mice infused with Ang II, especially after the treatment with α-galactosylceramide (αGC), a synthetic glycolipid that activates iNKT cells *via* CD1d. That correlated with increased incidence of AAA. Histopathological, immunofluorescent staining and RNAseq results also showed more severe infiltration by inflammatory cells in the Ang II+ αGC group (134). Interestingly, opposite results were found in another study, where activation of iNKT cells by αGC attenuated Ang II-mediated AAA in obese ob/ob mice *via* induction of anti-inflammatory macrophage polarization (135). Overall this points out to possible iNKT role in AAA development but complimentary “loss-of-function” experiments are still missing.

## Adaptive immunity

### T cells

T cells represent a key arm of adaptive immunity and are composed of CD4^+^TCRβ^+^ (helper) and CD8^+^TCRβ^+^ (cytotoxic) subsets. Depending on environmental cues CD4 T cells can differentiated toward Th1, Th2, Th17, Th22, regulatory T (Treg, CD4^+^FoxP3^+^CD25^+^) and more recently described Tfh lineages (136), most of which have been found in AAA (93, 105, 137). T helper subsets are characterized by the production of subset-specific cytokines impacting the inflammatory environment at the site of inflammation (138). Degradation of ECM proteins such as elastin and collagen progressing during AAA development is accompanied by CD4^+^ T cells infiltration (116). Recent study utilizing RNAseq on sorted “bulk/conventional” CD4 T cells revealed that CXCR6/CXCL16 axis is necessary for the recruitment of CD4 T cells to AAA. CD4 T cells were shown to produce GM-CSF, which in turn controls the recruitment and polarization of pro-inflammatory monocytes to the aortic wall through upregulation of CCL2 and activation of IRF5 (interferon regulatory factor 5) (139).

#### Th subsets: Th1 and Th2

Th1 cells are characterized by production of IFNγ, which plays a pro-inflammatory role in atherosclerosis (140–142). In AAA, however, the role of IFNγ is not clearly defined. Early studies showed that administration of recombinant IFNγ into mice lacking CD4^+^ T cells promotes aneurysm development (143). However, IFNγ deficiency was also associated with augmented AAA in Ang II–induced mouse model, suggesting a protective role for this cytokine in AAA (7). The proposed mechanism suggests that IFNγ is a regulator of CXCL10 expression in AAA, which in turn controls the recruitment of protective effector T cells (7). However, CXCL10 can also attract NK cells, which are considered pathogenic in AAA because of the production of so-called “type 2” cytokines (IL-4, IL-5 and IL-13) in antigen-independent, innate immune mode manner. Moreover, the neutralization of IFNγ by antibodies did not protect mice from AAA (144). Overall while these observations put IFNγ as an important player in AAA, they warrant further studies of its role at different stages of this disease, mechanisms of its induction, cell specificity of IFNγR signaling as well as cell type specific mechanisms of IFNγ production.

Th2 helper subset is characterized by the production of “type 2 cytokines” such as IL-4 and IL-5; and in that capacity these cells are similar to ILC2 and NK cells. These cytokines contribute to the control of B cell activation and clonal expansion (136, 145). They were shown to suppress early atherosclerotic lesions, however IL-4 deficiency only slightly alters the course of the disease (146, 147). IL-5 deficiency was shown to accelerate atherosclerosis (148). However, in AAA Th2 cells producing IL-4 and IL-5 were suggested to be pathogenic, particularly due to the ability to induce VSMC apoptosis (149, 150). The shift from Th1 to Th2 was associated with AAA augmentation (151). In humans, however, large AAA were characterized by Th1 cytokines profile whereas Th2 response was a predominant in patients with small aneurysms (152). The difficulty to assign a specific role for Th2 cells in AAA is related to the fact that type II cytokines can be also produced by NK cells and ILC2 (153, 154). The specific cellular source of type 2 cytokines had not been explicitly studied in AAA and future studies addressing cell specificity will be important.

#### Th17 cells

Th17 helper subset is regulated by the transcription factor RORγt and known to produce characteristic cytokines IL-17A, IL-17F and IL-22. Th17 cells are dependent on IL-23, IL-6 and IL-1β cytokines derived from myeloid and epithelial cells (155). Th17 cells play a pro-inflammatory, disease-promoting role in many inflammatory pathologies, including atherosclerosis (156–158) and had been implicated to AAA. IL-17A genetic deletion in elastase model of AAA attenuated the disease development and limited inflammatory cell infiltration (8). Similar phenotype was also observed in the Ang II-infusion model, where genetic and pharmacological neutralization of IL-17 or use of RORγt antagonist limited the disease (91, 159). Conversely, SOCS3 (suppressor of cytokine signaling 3) overexpression and reduction of IL-17A expression accelerated AAA (160). It is important to note that SOCS3 has multiple downstream targets beside IL-17A, for instance IL-10, which has its own, protective function in AAA. As Th17 cells expansion is driven by IL-23, its genetic and pharmacological ablation mitigated AAA, which was associated with reduced IL-12p40 production and lowered MMP expression (94).

Overall, more mechanistic studies better dissecting cell type specific responses are needed to elucidate the relative contribution of Th1 versus Th2 versus Th17 or other subsets of CD4 T cells in comparison with other cell types producing similar cytokines in AAA (161).

#### Regulatory T cells

Tregs are professional suppressors of immune and inflammatory responses known to inhibit the activation of other T cells and innate immune cells, thereby controlling the inflammation, autoimmunity, and anti-tumor immunity (162). Tregs had been detected in aortic tissue and their protective role in atherosclerosis had been demonstrated in multiple studies (163, 164). Tregs were also implicated to AAA pathogenesis by suppressing inflammatory cell accumulation (mainly macrophages and T cells) and proinflammatory molecules expression including CCL2, IL-6 and ICAM-1 (165, 166). Prostanoids and eicosanoids are essential inflammatory mediators associated with AAA development, and cyclooxygenase COX2, an enzyme regulating the conversion of arachidonic acid to prostanoids and eicosanoids, expression is upregulated in patients with AAA (167). Tregs can suppress COX2 expression by myeloid cells, thereby limiting AAA (168).

T cell co-inhibitory molecule cytotoxic T lymphocyte associated antigen-4 (CTLA4) is known to act as a potent negative regulator of immune responses (169). Overexpression of CTLA4 in CTLA-4 transgenic *Apoe^-/-^* mice fed with WD and infused with Ang II limited AAA incidence by 66%, reduced the diameter of abdominal aorta and mortality by 26% (110). These effects led to lowered number of accumulated CD4 T cells and downregulated expression of CD80 and CD86 (ligands for CTLA-4) on CD11c^+^ dendritic cells in lymphoid tissues. CD11c depletion led to reduced accumulation of macrophages and CD4 T cells, attenuating aortic inflammation, preserved vessel integrity, and decreased AAA and aortic rupture (110). In atherosclerosis Tregs in the aorta were shown to lose their suppressing anti-inflammatory properties converting to pro-inflammatory subsets. The conversion was mediated by the environment in atherosclerotic plaque characterized by local hypoxia, dyslipidemia and overproduction of pro-inflammatory cytokines. During atherosclerosis development Tregs were shown to lose FoxP3 (Treg specific transcription factor) expression, thereby switching to exTregs and upregulating transcription factors typical for other Th subsets, for instance to Th1 or Tfh (follicular helper cells) (170–173). It remains to be determined whether such conversion also takes place in AAA.

Pharmacological treatment with statins has been widely used in CVD, in part due to their immunomodulatory properties. Simvastatin and Treg depletion with anti-CD25 antibody in *Apoe^-/-^* mice subjected to Ang II infusion lowered the incidence and severity of AAA accompanied by reduced VSMC apoptosis and ROS production in the aortic wall (174). In patients with AAA receiving simvastatin, the levels of Ang II signaling marker caveolin-1 and Nrf2 activation were decreased (175), while protective eNOS expression was increased (176), suggesting its beneficial effect in AAA.

#### T follicular helper cells and T follicular regulatory helper cells

T follicular helper cells (Tfh) are localized in the germinal centers of secondary lymphoid organs where they regulate antibody class switching in B cells thereby controlling humoral immunity. Tfh are characterized by the expression of transcriptional factor Bcl-6 (B cell lymphoma 6) as well as CXCR5 and PD-1 (programmed death-1) (177, 178). A circulating subpopulation is characterized by CXCR3 and CCR6 expression and can be divided on cTfh1 (CXCR3^+^CCR6^−^; producing IFNγ), cTfh2 (CXCR3^−^CCR6^−^; secreting IL‐4, IL‐5, and IL‐13), and cTfh17 (CXCR3^−^CCR6^+^; producing IL‐17A and IL‐22) subsets (179). Tfh cells have been implicated to the regulation of autoimmune and inflammatory diseases including CVD. Their presence had been detected in the aortic wall (180) and CXCR3+ Tfh cells were found elevated in atherogenic environment (177, 178). Moreover, decreased frequency of cTfh1 and increased frequency of cTfh2 and cTfh17 had been described in patients with atherosclerosis compared to healthy controls (179). The genetic ablation of Bcl-6 in CD4^+^T cells slightly reduce atherosclerotic plaque size in *Apoe^-/-^* mice (178).

T follicular regulatory helper cells (Tfr) are Tfh cells that also express FoxP3 as well as IL-10 and TGFβ. Tfr cells were shown to suppress the activation of Tfh cells upon adoptive transfer to *Apoe^-/-^* mice causing marked decrease of Thf population along with atherosclerotic plaque size (181). However, the role of these cells in AAA is yet to be investigated.

### CD8 T cells

Much less is known about the contribution of CD8 cytotoxic T cells to AAA development. Early studies found CD8^+^CD28^−^ IFNγ producing T cells in AAA tissue and in circulation. Besides, a population of CD8 T cells lacking CD27 (that allows accumulation of CD8 T cells in tissues) was detected in human AAA lesions but not peripheral blood, suggesting a potential unique role of this subset of CD8 T cells in AAA (182). More recently, the role of CD8 T cells was assessed in the elastase model of AAA utilizing *Cd8^-/-^* animals and transgenic CD8 T cells. The study suggested that CD8, but not CD4 T cell derived IFNγ activates MMP9 and MMP2, thereby enhancing AAA development (183).

### γδ T cells

γδ T cells is a subset of T lymphocytes that can directly recognize antigen without APC and produce IL-17 and IFNγ. γδ T cells were detected in atherosclerotic aortas and were suggested to regulate neutrophil activation in IL-17 dependent manner (184). In humans, no difference in proportions of CD4, CD8, and γδ1^+^ T cells were detected between aneurysm tissue and PBMCs (185). At the same time γδ2^+^ T cells were found in greater numbers in aorta, and the frequency of Tregs was significantly lower in AAA compared to PBMCs (185). The number of CXCR5 expressing Vδ2^+^ T cells was significantly increased in aneurysm tissue compared to normal aorta or PBMCs from patients with aneurysm. Moreover, the frequency of IL-17A^+^ cells in AAA was significantly higher among γδ2^+^ T cells compared to CD4 or CD8 T cells. Importantly, IL-17A-producing γδ2^+^ T cells were found only in the aortic tissue, implying their potential role in the progression of AAA (185). In experimental model, γδ T cell deficiency inhibited the inflammatory response in the aorta and attenuated AAA, suggesting overall pro-inflammatory AAA-promoting role for γδ T cells (184).

### B cells

B cells represent another key arm of adaptive immunity performing their functions *via* antibody and cytokine production as well as antigen presentation. Two types of B cells had been described: B1 and B2 (186). B1 cells originate from fetal liver and in adult organisms mostly reside in the abdominal cavity. They are active producers of IgM antibody with predominant specificity to different components of bacterial products and phospholipids; their activation is mostly T-cell independent. B1 cells control atherosclerosis by the production of LDL specific IgM antibodies, which suppressed inflammatory macrophages polarization and foam cell formation (186). B2 cells develop in bone marrow and differentiate to antibody-producing plasma cells upon antigen exposure and help from T cells. B2 cells produce different flavors of antibodies, including IgG, IgA and IgE, as well as cytokines IL-10 and IL-6 (186), and can further promote Th1 and Th17 cell responses. IgE was shown to activate CD4 T cells and macrophages through FcϵR1 receptor recognizing IgE (4). In atherosclerosis, high fat diet (HFD) was shown to enhance the activation of T cells by augmenting cDC production, a mechanism in part mediated by B cell derived GM-CSF (186). In addition, atherosclerotic plaque is a source of endogenous TLR ligands that might promote B cell activation (186).

The role of B cells in AAA had been also suggested (187, 188). B cells had been detected mostly in adventitia in both human and mouse AAA (5, 187, 189). Ablation of B cells by knockout of muMT (IgG heavy chain) or cell depletion by anti-CD20 reduced AAA development in elastase and Ang II infusion models, which was associated with an increased presence of Tregs (187, 189, 190). B cell-deficient muMT mice were presented with reduced expression of MMP9. Furthermore, inhibition of key B cell receptor signaling molecule Syk suppressed AAA growth, reduced inflammatory response and limited immunoglobulin deposition in AAA (190). This overall suggests that B2 cells promote AAA. B cell accumulation in CaCl_2_-induced AAA at various stages of the disease progression does not significantly change during the disease progression, however, IgG and IgM had been detected in AAA with a peak at 1 week after the CaCl_2_ perfusion (190). Interestingly, B cell activation and production of autoantibodies such as anti-Hsp70, anti-Hsp65, or anti-AT1R occurs in humans and rodent models of hypertension (191), suggesting a potential role of Ang II in B cell activation. BAFF, a member of the tumor necrosis factor family of cytokines, drives the differentiation of B cells and is a critical survival factor for mature B cells. Recent studies demonstrated that blockade of BAFF receptors in elastase perfusion model attenuated AAA (170). The antagonist of BAFF depleted most of mature B cell subsets in spleen and circulation, decreased infiltration of B cells to the aorta, along with proinflammatory macrophages, and reduced number of apoptotic cells in AAA. The study also reported that in AAA tissue, B cells and macrophages were found in close contact (188, 190), suggesting a possible role of B cell-macrophage communication in AAA pathology. Future studies will be needed to elucidate the mechanisms regulating B cell activation in AAA, role of specific antibodies, including IgA as well as contribution of B1 cells and IgM to AAA pathogenesis.

Taken together, most of the immune subsets have well established or suggested function in AAA which is further summarized in **Table 1**, along with the information of genetic or pharmacological tools helpful to ascertain the role of various cells in AAA promotion or inhibition.

**Table 1:** The role of immune cells in AAA.

Cell type	Target	Intervention	Effect on AAA	AAA model	Ref
**Neutrophils**	Neutrophil depletion	Anti-PMN		Elastase	(19)
	Inhibition of NETosis	Losartan		*Ex vivo*	(38)
		Cl-amidine, YW3-56		Elastase	(46, 47)
**Monocytes /Macrophages**	Ly6C^high^ monocytes	Ang II infusion	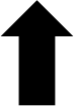	Ang II	(6, 16)
		Angiopoietin-2		Ang II	(64)
	CCR2	*Ccr2*^-/-^		Ang II	(65)
	Ly6C^low^ monocytes	*Nr4a1*^-/-^	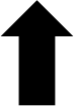	Ang II	(72)
	CD11b^+^ cells	*Itgam*^-/-^		CaCl_2_	(66)
	MMP9	*Mmp9*^-/-^		CaCl_2_	(87)
	MMP2	*Mmp2*^-/-^		CaCl_2_	(87)
	TNFα	*Tnf*^-/-^, TNFα antagonist (infliximab)		CaCl_2_	(89)
	TNFR1	*Tnfr1*^-/-^		Ang II	(90)
	Macrophage exosomes	GW4869 (exosome inhibitor)		CaPO_4_	(96)
	IL-23	*Il123*^-/-^, anti-IL-23 antibody		Ang II	(94)
	Netrin 1	*Ntn1*^-/-^ in hematopopietic cells		Ang II	(95)
	NLRP3, ASC or Caspase-1	NLRP3^-/-^, ASC^-/-^ or caspase-1^-/-^		Ang II	(83)
**Macrophages/Neutrophils/DC**	IL-1	*Il1β*^-/-^, anti IL-1*β*		CaCl_2_	(81)
		*Il1β*^-/-^, *Il1r*^-/-^	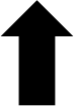	CaCl_2_	(82)
**Macrophages/DC**	IL-12p40	Anti IL-12p40 antibody		Elastase	(75)
			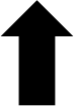	Ang II	(94)
**Monocyte/Macrophages/** **CD4T Th1**	TLR4	*Tlr4*^-/-^		Ang II	(80)
		Eritoran (drug), TLR4 signaling suprresion		Ang II	(80)
**Macrophages/ Treg**	TGF-β	*Tgfbr^flox/flox^* Acta2-CreER (smooth muscle specific)	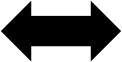	Ang II	(103)
		AntiTGFβ antibody	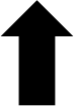	Ang II	(103)
	IL-10	rIL-10 infusion		Elastase	(100)
		IL-10 systemic induction with minicircle vector transfection		Ang II	(101)
**Monocytes/Macrophages/** **Neutrophils/** **CD4T cell Th17**	IL-6	*Il6*^-/-^		Ang II	(93)
**Dendritic cells**	CD11c+ cells	*Cd11c*DTR		Ang II	(112)
**Mast Cells**	IgE	*Ige*^-/-^, anti-IgE		Ang II	(116, 117)
				CaCl_2_	
	Meprin α	*Meprin1a*^-/-^		Ang II	(117)
**Th1/CD8/NK/ILC1/γδ T cells**	IFNγ	*Ifng*^-/-^	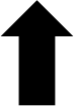	Ang II	(7)
		Anti IFNγ	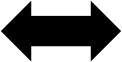	Ang II	(150)
**CD4T Th2/ILC2/NK**	IL-4	Anti IL-4	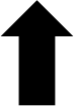	Elastase	(150)
**Th2/NK/ILC**	IL-5	Anti IL-5	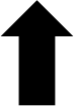	Ang II	(149)
**Th17/ILC3**	IL-17A	*Il17a*^-/-^		Elastase	(8)
		*Il17a*^-/-^ and anti IL-17A		Ang II	(8, 159)
**Tregs**	CTLA4	CTLA-4 Tg		Ang II	(169)
	CD25	anti CD25(PC61) antibody		Ang II	(174)
**CD8 T cells**	CD8	*Cd8*^-/-^		Elastase	(183)
		*Cd8*Tg	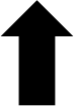	Elastase	(183)
**γδ T cells**	γδ Tcells	*γδTcr^-/-^*		Elastase	(184)
**B cells**	B2 cells	muMT^-/-^		Elastase	(190)
		Anti CD20		Ang II	(190)
		Anti BAFF		Elastase	(188)

## Mechanisms that regulate immune cells in AAA

**Figure 2:**
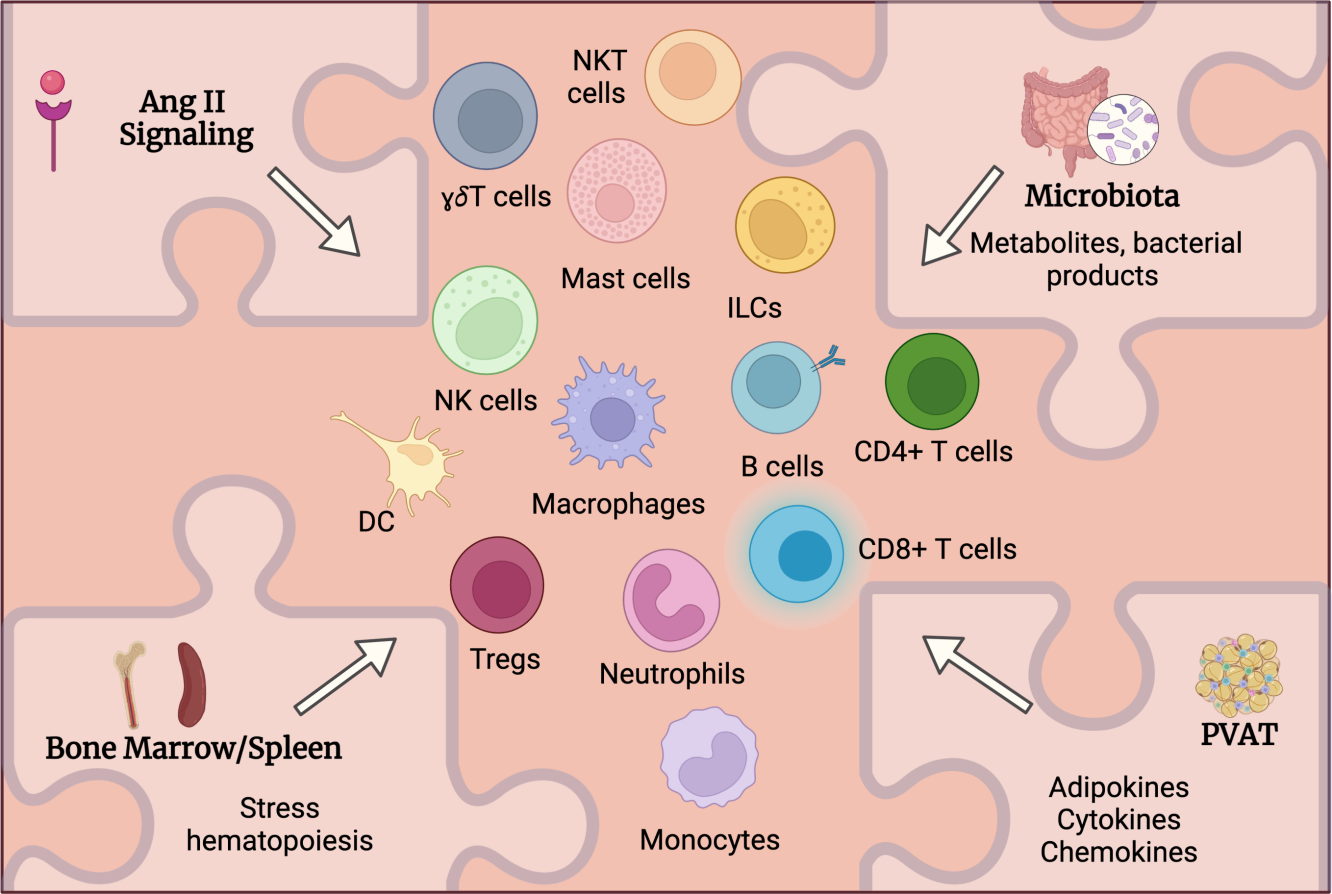
Potential mechanisms regulating immune cells activation in AAA. Multiple factors may systemically and locally regulate immune cells activation in AAA. Angiotensin (Ang) II receptors are expressed on various immune cells and can directly regulate their activation and function. Alteration of intestinal barrier will impact microbiota composition and function leading to changes in circulating metabolites and microbial products that in turn may regulate immune cell activation in AAA. Ang II as well as microbiota-derived products may stimulate immune cell mobilization from bone marrow and spleen. Perivascular adipose tissue (PVAT) may contribute to the inflammation in the aortic wall *via* the production of adipokines, cytokines and chemokines regulating immune cells accumulation in PVAT.

While various immune cells had been detected in aortic tissue and multiple study attempted to address the mechanistic role of these cells in AAA, the upstream mechanisms controlling immune cells activation and accumulation in AAA are still under investigation. Despite previously established roles of listed below factors in immune cell activation in other diseases including CVD, one can hypothesize and extrapolate it to AAA to suggest how these factors influence immune activation and function in AAA and where spatially the activation of immune cells occurs (**Figure 2****)**.

### Angiotensin II

High blood pressure is one of the key risk factors for AAA. RAS (Renin-Angiotensin-Aldosterone System) controls vasoconstriction and blood pressure. Moreover, RAS was also implicated in the regulation of cell growth and vascular wall integrity influencing many cellular processes (192). Ang II is one of the key enzymes of RAS. Ang II acts through its receptors AT1R and AT2R to regulate cardiovascular remodeling. Ang II also shares some of the signaling pathways with growth factors, promoting growth of cardiac myocytes, fibroblasts and vascular smooth muscle cells (VSMCs) *via* MAPKKK pathway (193, 194). AT1Rs are expressed by vascular, endothelial cells and various immune cells (13) suggesting Ang II involvement in their activation. Multiple studies demonstrated the effect of Ang II on immune cells. For example, Ang II was shown to induce a pro-inflammatory program in THP-1 macrophages *in vitro* (195). Moreover, interaction between Ang II and core clock gene Rev-erbα in macrophages had been proposed, which through the AT1R/LXRα pathway was implicated to the control of MMP9 expression (196). Furthermore, Ang II was shown to control not only mature immune cells but also hematopoietic stem and progenitor cells (HSPC) leading to enhanced myeloid differentiation and myeloid cells production (197).

Recent work identified cytokine-dependent mechanisms that cooperate with Ang II to induce stress myelopoiesis and AAA (198). Specifically, IL-27R signaling was shown to potentiate the response of HSPC in the bone marrow to Ang II. The ablation of IL-27R in mice infused with Ang II protected them from AAA (198). Mitigation of aneurysm development was associated with blunted accumulation of myeloid cells in the aorta due to attenuation of Ang II-induced HSC expansion. Mechanistically, IL-27R signaling was required to induce transcriptional programming to overcome HSC quiescence and increase differentiation and output of mature myeloid cells in response to stress stimuli to promote their accumulation in the diseased aorta (198). It is conceivable that other cytokines (such as IL-1 and IFNγ) may also conspire with Ang II to enhance emergency myelopoiesis from BM during AAA development.

Spleen was shown to play an important role as a reservoir for extramedullar hematopoiesis (199), that had been previously linked to atherosclerosis development (200). The mobilization of both Ly6C^high^ and Ly6C^low^ monocytes from the spleen in response to Ang II had been reported (16). B cells were suggested to regulate early monocyte mobilization and promote macrophage accumulation in the AAA through mediation of extramedullary hematopoiesis. Splenectomy prior to Ang II infusion inhibited early monocyte mobilization and protected from AAA (16).

Cells of adaptive immunity also express ATRs. It has been reported that Ang II stimulation activated inflammatory phenotype in T cells and facilitates their infiltration to adventitia and perivascular adipose tissue (PVAT) as well as into the heart (191). The modulation of adaptive immune activation in hypertension has been attributed to target organ oxidative stress and was suggested to be sex dependent (191). Although effects of Ang II on T cells have been explored, the role of Ang II in the regulation of B cells in AAA is poorly understood despite AT1R being expressed on B cells.

Ang II was suggested to affect the composition of gut microbiota, and therefore a possible crosstalk between microbiota-inducing cytokines and Ang II during AAA development should be taken into the consideration (**Figure 2**). Systemically, Ang II was shown to modify plasma and fecal metabolites in conventionally raised mice vs germ-free (GF) animals, suggesting the role of Ang II in the regulation of intestinal epithelial cells and microbiota (201), possibly in a sex-dependent manner (201). Despite multiple studies convincingly demonstrating an important role of Ang II in AAA (13), additional work is needed to better understand the cell specificity of Ang II signaling in this disease.

### Microbiota in CVD

Diet, inflammation, aging and increased bone marrow (BM) myeloid cell output all contribute to the development of CVD (102, 202). Microbiota is a common facilitator for processing and metabolizing food, inducing inflammation and regulating BM output. A connection between unhealthy diet, alterations in bacterial composition in the intestine and CVD has recently emerged (203). The relationship between dysbiosis and obesity has been suggested, supporting the emerging view that gut microbiota contribute to metabolic disease by modulating host metabolism (202, 204–206). Germ free (GF) mice are resistant to diet-induced obesity, with some mechanisms of microbiota modulating inflammation and lipid metabolism suggested. GF mice are characterized by reduced adipose tissue inflammation, while the presence of gut microbiota increases macrophage content in the fat with a polarization toward pro-inflammatory phenotype (207, 208). Metabolic alterations that contribute to atherosclerosis and possible contribution of gut microbiome to this disease development *via* altered production of microbial- or food-derived metabolites have been also reported (208–212), however more mechanistic studies are needed to demonstrate causative rather than correlative effect.

The composition of the diet was shown to regulate barrier function of the intestine. Hence, intestinal permeability and alterations in the microbial community in the gut are affected by high fat, high carbohydrate “Western diet” and can cause translocation of bacterial products and metabolites, further impacting CVD development (213–218). Moreover, microbiota-derived factors can modify activation state of intestinal epithelial cells (IEC) and immune cells. For instance, high fiber diet induced favorable changes in microbiota, and played a protective role in the development of atherosclerosis by controlling acetate (short chain fatty acid) production. Its effects were accompanied by downregulation of *Egr1*, a master regulator gene involved in cardiac hypertrophy, cardiorenal fibrosis, and inflammation (210). On the other hand, dietary serves as a substrate for microbiota-catalyzed overproduction of di- and tri-methylamines. Trimethylamine-N-oxide (TMAO) is a metabolic derivative of L-carnitine and choline; found to be upregulated in patients with CVD, and its serum levels correlate with higher risk of myocardial infarction and atherosclerosis development (211, 219).

Microbiota and its products also has been shown to influence hematopoietic stem cell (HSC) differentiation and BM output (220). At the same time, WD was suggested to impact epigenetic reprogramming of granulocyte and monocyte precursor cells in NLRP3 inflammasome dependent manner, skewing bone marrow cell differentiation toward myeloid lineages and enhancing so-called “trained immunity” in atherosclerosis. This reprogramming is maintained even after the switch to chow diet, showing long lasting “trained immunity” induced by WD along with enhanced production of myeloid cells which are later on recruited into CVD lesion sites (52).

Single-cell RNA sequencing of human AAA tissues revealed increased expression of histone demethylase JMJD3 in aorta infiltrating monocyte and macrophages, resulting in reduction of repressive histone methylation H3K27me3 marks on promoters of inflammatory genes and concomitant upregulation of inflammatory gene expression. *Jmjd3* expression was shown to be controlled by IFNβ/JAK/STAT pathway and led to NF-κB-dependent induction of inflammatory gene transcription in aorta-infiltrating macrophages contributing to vascular inflammation (221). These results suggest that epigenetic modifications could play a modulatory role in the regulation of inflammatory environment in AAA.

#### Microbiota and AAA

While many studies are focused on the connection between alterations of microbiota and atherosclerosis, its contribution to AAA is far less understood. Change in gut microbiota composition is linked to hypertension in rodents and humans (202, 207, 222). The relationship between microbiota diversity and AAA severity in humans has been recently reported, and changes in microbiota composition correlated with AAA presence and size (223). Specifically, a decrease in relative abundance of Bacteroidetes and increased relative abundance of Proteobacteria had been reported in patients with AAA (223). The relation between AAA size and α-diversity index was inverse, and severity had a positive correlation with increased relative abundance of Enterobacteriaceae and decreased abundance of Veillonellaceae (223). The reduction of the Verrucomicrobia (particularly represented by *Akkermansia)* was detected in mice infused with Ang II (224).

Germ-free (GF) mice infused with Ang II were characterized by lower neutrophil infiltration into the aorta, attenuated cardiac and kidney inflammation, fibrosis, and systolic dysfunction (207). Accordingly, attenuated leukocyte adhesion, lowered infiltration of Ly6G^+^ neutrophils and Ly6C^+^ monocytes into the aortic wall, limited endothelial dysfunction and reduction of blood pressure were observed in GF mice subjected to Ang II infusion, indicating possible contribution of gut microbiota to immune cells activation in response to Ang II (207). A link between normal vascular function and microbiota was directly proven by microbiota depletion (225). When young GF mice were analyzed for vascular contractility and structure, males and females showed differential response: males showed a marked decrease in contraction of arteries and increased vascular stiffness, while females showed hypertrophic remodeling. Also, ROS generation by neutrophils was blunted in female GF mice and exacerbated in male GF mice (225).

While several microbiota-derived products and metabolites had been implicated in the regulation of immune cells activation in CVD as described above, most of the studies to date had been focused on atherosclerosis. It remains to be determined whether similar mechanisms are also specifically involved in the pathogenesis of AAA. Here we will briefly discuss microbial metabolites and their role in CVD and possible contribution to AAA.

##### LPS

LPS is an obligatory component of gram-negative bacteria wall. Elevated levels of LPS can be detected in the circulation as a result of altered gut barrier and increased gut permeability and alteration of microbiota composition. Unhealthy diets can induce gut dysbiosis and alter gut barrier function, thereby contributing to elevated LPS in the circulation. LPS promotes activation of myeloid cells *via* TLR4/MyD88 pathway, systemic inflammation and monocyte infiltration to vascular wall (226–228). The effect could be further exacerbated by combined action with other cardiovascular disease modifying factors. Hence, LPS in combination with oxLDL was shown to induce NLRP3 inflammasome activation and IL-1β production by macrophages that contribute to atherosclerosis development (52). LPS induces various pro-inflammatory cytokines production including IL-6, TNF as well as Osteopontin (Spp1) by macrophages (52, 227, 229, 230). Moreover, LPS together with oxLDL was shown to inhibit cholesterol transporters ABCA1 and ABCG1, which in turn affects reverse cholesterol transport (231). Furthermore, LPS together with TMAO had been demonstrated to enhance *Spp1, Il1b*, and *Cd36* gene expression in the aorta (229).

*In vitro* study comparing monocyte-derived macrophages from AAA patients and matched controls showed limited response to LPS due to TLR4 cytosolic internalization, which may be a sign of diminished inflammatory responsiveness, but also may reflect an excessive LPS signaling which resulted in “LPS tolerance” (232).

##### TMAO

TMAO (trimethylamine-N-oxide) is produced in the liver from choline and L-carnitine dietary-derived metabolite TMA (trimethylamine) whose production itself requires gut microbiota. TMAO production has been associated with the presence of specific bacterial taxa in the gut, for example *Prevotella spp* (202, 211, 233). Elevated serum TMAO was found in individuals with CVD and was implicated into atherosclerosis and thrombosis (218, 229, 234–240). In experimental models L-carnitine or choline supplementation to mice led to the upregulation of TMAO and augmented atherosclerosis acting by reducing *in vivo* cholesterol reverse transport and modifying microbiota (202, 233). Moreover, no upregulation of TMAO was detected in GF mice despite L-carnitine supplementation, thus no augmentation of atherosclerosis was found (211), implying an important link between intestinal microbiota, TMAO production and CVD. TMAO had been also implicated into the regulation of platelet hyperreactivity and thrombus formation and these parameters were reduced in the absence of microbiota in GF mice (208).

The bacterial fermentation products such as lactate and acetate had been shown to regulate storage and metabolism of lipids in intestinal epithelial cells *via* control of β-oxidation and PPARα pathways (209). However, not all studies demonstrated a notable effect of microbial products on CVD. For example, one study reported that choline supplementation or WD feeding in conventionally raised and GF mice led to a minor dysbiosis in mice, but the effects on atherosclerosis were only driven by cholesterol levels in plasma (212). The role of TMAO in AAA has been recently suggested. TMAO added to drinking water promoted AAA development in Ang II and CaCl_2_ mouse models (241). This was accompanied by heightened elastin degradation and upregulation of ROS, MMP-2 and 9 and senescence markers in the aorta (241).

##### Bile acids

Multiple members of gut microbiota including *Clostridium, Bifidobacterium* and *Lactobacillus* participate in bile acid metabolism through bacterial bile-salt hydrolase (BSH) activity, necessary for the bile acid deconjugation and formation of free bile acids and taurine residues (242). Decreased bile-salt hydrolase in dysbiotic conditions had been linked to enhanced foam cell formation by upregulation of hepatic FXR (Farnesoid X Receptor) and inhibition of Cyp71a (cholesterol 7 alpha-hydroxylase) and LXR, thus promoting cholesterol accumulation within the liver, intestinal cells and plaque macrophages (243). Bile acids such as deoxycholic acid (DCA), signal through G protein-coupled BA receptor 1 (TGR5), causing the activation of macrophages and the production of inflammatory cytokines. Interestingly, it was suggested that low concentrations of secondary BA may have anti-inflammatory effects, whereas high concentrations are clearly pro-inflammatory (244). Ang II infusion to conventional raised mice resulted in upregulation of taurodeoxycholate and taurodeoxycholic acid in feces, while no changes had been observed in GF mice (201). Taurodeoxycholate has been shown to lower blood pressure in rats (201), which suggests that microbiota may also exerts beneficial effects in the host as a homeostatic mechanism.

##### Short chain fatty acids (SCFA)

SCFA including propionate, butyrate and acetate are produced by gut bacteria from dietary fiber. These metabolites can regulate inflammation in intestinal macrophages by signaling through G-protein coupled receptors or by inhibiting histone deacetylases (244). SCFA also contribute to the expansion of Tregs and IL-10 secretion in colon (244). The reduction of butyrate producers such as *Eubacterium* and *Roseburia* in atherosclerosis had been reported and was associated with increased adhesion of monocytes to the inflamed endothelium, promoting plaque development (243). Propionate administration in hypertensive *Apoe^-/-^* mice lowered systemic inflammation and attenuated hypertension, vascular dysfunction, atherosclerosis and fibrosis. The role of SCFA in the inflammatory environment in AAA remains to be experimentally tested directly. Dietary supplementation of propionate has been suggested for AAA patients, but its actual benefits remain to be determined (217).

As the relevance of microbiota to CVD emerges the number of identified metabolites will continue to grow. For example, recently Nemet et al. identified the correlation between plasma metabolite phenylacetylglutamine (PAGln) and severity and outcomes of myocardial infarction and stroke (245). PAGln enhanced platelet activation and thrombosis signaling through G-protein coupled receptors, such as α2A, α2B and β2-adrenergic receptors (245).

## Role of perivascular adipose tissue in AAA development

Perivascular adipose tissue (PVAT) is adipose tissue that surrounds the vessels as a distinct layer. As any other adipose tissue PVAT is composed of white and brown adipocytes, and fibroblasts (234). It is infiltrated by multiple immune cells (234) and heavily innervated (246, 247). In pro-inflammatory environment or under the conditions of lipid overload, adipocytes become activated and produce pro-inflammatory cytokines (including TNF and IL-6) and adipokines (such as leptin) which further facilitate immune and VSMC cell activation (234, 248–250). Increased adiposity and lipid deposition is associated with the shift toward white adipocytes and heightened accumulation of pro-inflammatory immune cell types (251), for example pro-inflammatory adipose macrophages (252). In obesity the inflammation in the visceral adipose tissue can be mediated by skewing T helper response from Tregs towards pro-inflammatory T helper subsets, with loss of a unique population of Ly6C^+^ Tregs normally localized in “lean” adipose tissue (253). Therefore, increase in white adipose tissue accompanied by low-grade inflammation, PVAT may contribute to AAA development given that it contains higher number of immune cells compared to healthy aortic wall (254). PVAT is also implicated in the control of vascular tone, however both anti- or pro-contractile effects had been described (234, 255–259). Moreover, PVAT was also shown to play a critical role in vascular regulation by local secretion of RAS components, including Ang II, as well as production of other entities which regulate vessels, blood pressure and inflammation, including leptin, IL-6, catecholamines and prostanoids, resistin and adiponectin (234, 259, 260). Angiotensin and aldosterone are both present in PVAT tissue and modulate endothelial dysfunction as well as immune cell infiltration (261).

The role of Ang II receptor signaling in adipocytes had been recently suggested. Adipose tissue transplantation from *Apoe^-/-^At1r^-/-^* mice to *Apoe^-/-^* mice infused with Ang II attenuated aortic aneurysm formation, macrophage infiltration, osteopontin expression by macrophages and gelatinolytic activity in the abdominal aorta (255). Levels of ceramides in PVAT correlated with elevated accumulation of macrophages and T cells in human AAA (262). Indeed, both CD4 and CD8 T cells had been detected in human samples, and activated CD69^+^ CD4 T cells were present in higher numbers in PVAT than in AAA lesions and their accumulation was dependent on Ang II (152, 262). B1 cells were also detected in PVAT in human and mouse aorta samples (263). The effects of microbiota and circulating metabolites on PVAT that can further transpire to affect AAA are largely unknown and will remain a subject of future studies.

## Perspective

During the past decade the contribution of immune cells to the pathogenesis of AAA became evident and different immune cells had been found in AAA lesions. Some mechanistic studies provided evidence regarding the role of immune cells in AAA pathogenesis. However, the specific contribution of immune cell subsets remains poorly understood and warrants future studies using cell type specific knockouts and more physiologically relevant models. Remaining questions include the understanding of the dynamic of immune cell accumulation and their contribution at early or more advanced stages of the disease. For example, it would be very interesting to determine experimentally whether initial steps of AAA development are actually mediated by recruited neutrophils (and monocytes) or they are only responders to the injury of the aorta generated by other factors. The spatiotemporal changes in cell lineage plasticity (for macrophages, neutrophils or VSMC) will be further addressed through in depth multiproteomic phenotyping and next generation single cell RNA sequencing. These approaches are also great for in depth mechanistic studies of samples derived from human AAA patients.

Better understanding of the mechanisms regulating immune cells activation and accumulation in AAA would provide an important knowledge for therapeutic interventions and likely will allow consideration of new preventive approaches. While the role of microbiota alterations had been implicated to the control of chronic inflammatory diseases including atherosclerosis, the field still gathers and catalogues data on the roles and mechanisms of microbiota action in AAA. A particular interest may represent a focus on the effect of diets and food additives that had been demonstrated to impact microbiota composition and function as well as affect immune cells and inflammatory responses. Future mechanistic studies focusing on interplay between microbiota, metabolites and immune cells during AAA initiation and progression will be of a great interest and potential translational importance.

Several models of AAA development in rodents had been developed and widely used in experimental studies, however they are not fully reflecting human pathology and frequently provide opposite results which are likely related to the nature and limitations of the models. The immune cell responses and requirements for specific immune subsets or mediators may vary between the models. It would be important to take into the account chronic inflammatory nature of AAA as well as contribution of systemic factors such as microbiome when experiments are designed and interpreted.

Overall, understanding of novel immune mediated mechanisms regulating AAA development and factors driving pathogenic immune cell activation will pave the road for novel therapeutics and preventive approaches in AAA and other CVDs, therefore representing an exciting area of research for future studies.

## Author contributions

AM-S prepared the Figures, AM-S and EK wrote the manuscript. All authors contributed to the article and approved the submitted version.

## Funding

The work was supported by NIH R01 HL133669, R01 HL149946 and NCI R01 CA273925 grants to EK.

## Acknowledgments

We thank Dr. S. Grivennikov for critical reading of the manuscript. Figures were created with BioRender.com.

## Conflict of interest

The authors declare that the research was conducted in the absence of any commercial or financial relationships that could be construed as a potential conflict of interest.

## Publisher’s note

All claims expressed in this article are solely those of the authors and do not necessarily represent those of their affiliated organizations, or those of the publisher, the editors and the reviewers. Any product that may be evaluated in this article, or claim that may be made by its manufacturer, is not guaranteed or endorsed by the publisher.

## References

[1] Cardiovascular diseases (CVDs). Available at: https://www.who.int/en/news-room/fact-sheets/detail/cardiovascular-diseases-(cvds) (Accessed July 16, 2021).

[2] WHO. Cardiovascular diseases (CVDs). WHO (2019). Available at: https://who.int/en/news-room/fact-sheets/detail/cardiovascular-diseases-(cvds) (Accessed July 05, 2020).

[3] Lu H Rateri DL Bruemmer D Cassis LA Daugherty A. Novel mechanisms of abdominal aortic aneurysms. Curr Atheroscler Rep (2012) 14:402–12. doi: 10.1007/s11883-012-0271-y PMC3436976 22833280

[4] Wang J Lindholt JS Sukhova GK Shi MA Xia M Chen H. IgE actions on CD4+ T cells, mast cells, and macrophages participate in the pathogenesis of experimental abdominal aortic aneurysms. EMBO Mol Med (2014) 6:952–69. doi: 10.15252/emmm.201303811 PMC4119357 24963147

[5] Dale MA Ruhlman MK Baxter BT. Inflammatory cell phenotypes in AAAs: Their role and potential as targets for therapy. Arter Thromb Vasc Biol (2015) 35:1746–55. doi: 10.1161/ATVBAHA.115.305269 PMC4514552 26044582

[6] Raffort J Lareyre F Clément M Hassen-Khodja R Chinetti G Mallat Z. Monocytes and macrophages in abdominal aortic aneurysm. Nat Rev Cardiol 2017 148 (2017) 14:457–71. doi: 10.1038/nrcardio.2017.52 28406184

[7] King VL Lin AY Kristo F Anderson TJ Ahluwalia N Hardy GJ. Interferon-gamma and the interferon-inducible chemokine CXCL10 protect against aneurysm formation and rupture. Circulation (2009) 119:426–35. doi: 10.1161/CIRCULATIONAHA.108.785949 PMC2765043 19139386

[8] Sharma AK Lu G Jester A Johnston WF Zhao Y Hajzus VA. Experimental abdominal aortic aneurysm formation is mediated by IL-17 and attenuated by mesenchymal stem cell treatment. Circulation (2012) 126:S38–45. doi: 10.1161/CIRCULATIONAHA.111.083451 PMC3448933 22965992

[9] Cheng J Zhang R Li C Tao H Dou Y Wang Y. A targeting nanotherapy for abdominal aortic aneurysms. J Am Coll Cardiol (2018) 72:2591–605. doi: 10.1016/J.JACC.2018.08.2188 30466517

[10] Yin L Zhang K Sun Y Liu Z. Nanoparticle-assisted diagnosis and treatment for abdominal aortic aneurysm. Front Med (2021) 8:665846/BIBTEX. doi: 10.3389/FMED.2021.665846/BIBTEX PMC8292633 34307401

[11] Bruijn LE Gómez CG van S Curci JA Golledge J Hamming JF Jones GT. A histopathological classification scheme for abdominal aortic aneurysm disease. JVS-Vascular Sci (2021) 2:260. doi: 10.1016/J.JVSSCI.2021.09.001 PMC8605212 34825232

[12] Golledge J. Abdominal aortic aneurysm: update on pathogenesis and medical treatments. Nat Rev Cardiol 2018 164 (2018) 16:225–42. doi: 10.1038/s41569-018-0114-9 30443031

[13] Sawada H Lu HS Cassis LA Daugherty A. Twenty years of studying AngII (Angiotensin II)-induced abdominal aortic pathologies in mice: Continuing questions and challenges to provide insight into the human disease. Arterioscler Thromb Vasc Biol (2022) 42:277–88. doi: 10.1161/ATVBAHA.121.317058 PMC8866209 35045728

[14] Cassis LA Gupte M Thayer S Zhang X Charnigo R Howatt DA. ANG II infusion promotes abdominal aortic aneurysms independent of increased blood pressure in hypercholesterolemic mice. Am J Physiol Heart Circ Physiol (2009) 296:H1660–5. doi: 10.1152/ajpheart.00028.2009 PMC2685354 19252100

[15] Cassis LA Huang J Gong MC Daugherty A. Role of metabolism and receptor responsiveness in the attenuated responses to angiotensin II in mice compared to rats. Regul Pept (2004) 117:107–16. doi: 10.1016/j.regpep.2003.09.008 14700746

[16] Mellak S Ait-Oufella H Esposito B Loyer X Poirier M Tedder TF. Angiotensin II mobilizes spleen monocytes to promote the development of abdominal aortic aneurysm in apoe-/- mice. Arter Thromb Vasc Biol (2015) 35:378–88. doi: 10.1161/ATVBAHA.114.304389 25524776

[17] Manz MG Boettcher S. Emergency granulopoiesis. Nat Rev Immunol (2014) 14:302–14. doi: 10.1038/nri3660 24751955

[18] Meher AK Spinosa M Davis JP Pope N Laubach VE Su G. Novel role of IL (Interleukin)-1β in neutrophil extracellular trap formation and abdominal aortic aneurysms. Arterioscler Thromb Vasc Biol (2018) 38:843–53. doi: 10.1161/ATVBAHA.117.309897 PMC5864548 29472233

[19] Eliason JL Hannawa KK Ailawadi G Sinha I Ford JW Deogracias MP. Neutrophil depletion inhibits experimental abdominal aortic aneurysm formation. Circulation (2005) 112:232–40. doi: 10.1161/CIRCULATIONAHA.104.517391 16009808

[20] Peshkova IO Aghayev T Fatkhullina AR Makhov P Titerina EK Eguchi S. IL-27 receptor-regulated stress myelopoiesis drives abdominal aortic aneurysm development. Nat Commun (2019) 10:5046. doi: 10.1038/s41467-019-13017-4 31695038 PMC6834661

[21] Vandestienne M Zhang Y Santos-Zas I Al-Rifai R Joffre J Giraud A. TREM-1 orchestrates angiotensin II–induced monocyte trafficking and promotes experimental abdominal aortic aneurysm. J Clin Invest (2021) 131:e142468. doi: 10.1172/JCI142468 PMC7810476 33258804

[22] Silvestre-Roig C Braster Q Ortega-Gomez A Soehnlein O. Neutrophils as regulators of cardiovascular inflammation. Nat Rev Cardiol (2020) 17:327–40. doi: 10.1038/S41569-019-0326-7 31996800

[23] Klopf J Brostjan C Neumayer C Eilenberg W. Neutrophils as regulators and biomarkers of cardiovascular inflammation in the context of abdominal aortic aneurysms. Biomed (2021) 9:1236. doi: 10.3390/BIOMEDICINES9091236 PMC8467789 34572424

[24] Soehnlein O Zernecke A Eriksson EE Rothfuchs AG Pham CT Herwald H. Neutrophil secretion products pave the way for inflammatory monocytes. Blood (2008) 112:1461–71. doi: 10.1182/blood-2008-02-139634 PMC3400540 18490516

[25] Kologrivova I Shtatolkina M Suslova T Ryabov V. Cells of the immune system in cardiac remodeling: Main players in resolution of inflammation and repair after myocardial infarction. Front Immunol (2021) 12:664457. doi: 10.3389/FIMMU.2021.664457 33868315 PMC8050340

[26] Chen F Eriksson P Hansson GK Herzfeld I Klein M Hansson LO. Expression of matrix metalloproteinase 9 and its regulators in the unstable coronary atherosclerotic plaque. Int J Mol Med (2005) 15:57–65. doi: 10.3892/ijmm.15.1.57 15583828

[27] Ma H Dong XF Cao XR Hei NH Li JL Wang YL. Pro-renin receptor overexpression promotes angiotensin II-induced abdominal aortic aneurysm formation in apolipoprotein e-knockout mice. Hum Gene Ther (2020) 31:639–50. doi: 10.1089/HUM.2019.124 31992084

[28] Moehle CW Bhamidipati CM Alexander MR Mehta GS Irvine JN Salmon M. Bone marrow-derived MCP1 required for experimental aortic aneurysm formation and smooth muscle phenotypic modulation. J Thorac Cardiovasc Surg (2011) 142:1567–74. doi: 10.1016/j.jtcvs.2011.07.053 PMC3627218 21996300

[29] Plana E Oto J Medina P Fernández-Pardo Á Miralles M. Novel contributions of neutrophils in the pathogenesis of abdominal aortic aneurysm, the role of neutrophil extracellular traps: A systematic review. Thromb Res (2020) 194:200–8. doi: 10.1016/J.THROMRES.2020.07.039 32788119

[30] Tall AR Westerterp M. Inflammasomes, neutrophil extracellular traps, and cholesterol. J Lipid Res (2019) 60:721–7. doi: 10.1194/JLR.S091280 PMC6446695 30782961

[31] An Z Li J Yu J Wang X Gao H Zhang W. Neutrophil extracellular traps induced by IL-8 aggravate atherosclerosis *via* activation NF-κB signaling in macrophages. Cell Cycle (2019) 18:2928. doi: 10.1080/15384101.2019.1662678 31496351 PMC6791689

[32] Soehnlein O Weber C. Myeloid cells in atherosclerosis: initiators and decision shapers. Semin Immunopathol (2009) 31:35–47. doi: 10.1007/s00281-009-0141-z 19238385

[33] Brandau A Ibrahim N Klopf J Hayden H Ozsvar-Kozma M Afonyushkin T. Association of lipoproteins with neutrophil extracellular traps in patients with abdominal aortic aneurysm. Biomedicines (2022) 10:217. doi: 10.3390/BIOMEDICINES10020217 35203427 PMC8869298

[34] Tang W Yao L Hoogeveen RC Alonso A Couper DJ Lutsey PL. The associations of biomarkers of inflammation and extracellular matrix degradation with the risk of abdominal aortic aneurysm: The ARIC study. Angiology (2019) 70:130. doi: 10.1177/0003319718785278 29945457 PMC6810681

[35] Aurelian SV Adrian M Andercou O Bruno S Alexandru O Catalin T. Neutrophil-to-Lymphocyte ratio: A comparative study of rupture to nonruptured infrarenal abdominal aortic aneurysm. Ann Vasc Surg (2019) 58:270–5. doi: 10.1016/J.AVSG.2018.11.026 30769065

[36] King AH Schmaier AH Harth KC Kumins NH Wong VL Zidar DA. Elevated neutrophil-lymphocyte ratio predicts mortality following elective endovascular aneurysm repair. J Vasc Surg (2020) 72:129–37. doi: 10.1016/j.jvs.2019.10.058 32037083

[37] Kordzadeh A Malietzis G Browne T Prionidis I Panayiotopoulos YP. Neutrophil to lymphocyte ratio (NLR) of five predicts 30-day morbidity in ruptured abdominal aortic aneurysms (rAAA): A retrospective cohort study. Int J Surg (2015) 15:45–8. doi: 10.1016/j.ijsu.2015.01.013 25641718

[38] Hofbauer TM Scherz T Ondracek A Müller J Mangold A Lang IM. Angiotensin-II enhances neutrophil extracellular trap formation in an AT1R and NADPH oxidase-dependent manner. Cardiovascular Res (2018) 144(1):S39. doi: 10.1093/cvr/cvy060.110

[39] Dahdah A Johnson J Gopalkrishna S Jaggers RM Webb D Murphy AJ. Neutrophil migratory patterns: Implications for cardiovascular disease. Front Cell Dev Biol (2022) 10:795784. doi: 10.3389/FCELL.2022.795784 35309915 PMC8924299

[40] de Waard V Bot I de Jager SCA Talib S Egashira K de Vries MR. Systemic MCP1/CCR2 blockade and leukocyte specific MCP1/CCR2 inhibition affect aortic aneurysm formation differently. Atherosclerosis (2010) 211:84–9. doi: 10.1016/J.ATHEROSCLEROSIS.2010.01.042 20197192

[41] Choke E Cockerill GW Laing K Dawson J Wilson WRW Loftus IM. Whole genome-expression profiling reveals a role for immune and inflammatory response in abdominal aortic aneurysm rupture. Eur J Vasc Endovasc Surg (2009) 37:305–10. doi: 10.1016/J.EJVS.2008.11.017 19111481

[42] Golledge J Clancy P Moran C Biros E Rush C Walker P. The novel association of the chemokine CCL22 with abdominal aortic aneurysm. Am J Pathol (2010) 176:2098. doi: 10.2353/AJPATH.2010.090416 20348247 PMC2861076

[43] Iida Y Xu B Xuan H Glover KJ Tanaka H Hu X. Peptide inhibitor of CXCL4-CCL5 heterodimer formation, MKEY, inhibits aortic aneurysm initiation and progression in mice. Arterioscler Thromb Vasc Biol (2013) 33:718. doi: 10.1161/ATVBAHA.112.300329 23288157 PMC4158029

[44] Hannawa KK Eliason JL Woodrum DT Pearce CG Roelofs KJ Grigoryants V. L-selectin-mediated neutrophil recruitment in experimental rodent aneurysm formation. Circulation (2005) 112:241–7. doi: 10.1161/CIRCULATIONAHA.105.535625 15998669

[45] Yan H Zhou HF Akk A Hu Y Springer LE Ennis TL. Neutrophil proteases promote experimental abdominal aortic aneurysm *via* extracellular trap release and plasmacytoid dendritic cell activation. Arterioscler Thromb Vasc Biol (2016) 36:1660. doi: 10.1161/ATVBAHA.116.307786 27283739 PMC4965335

[46] Meher AK Spinosa M Davis JP Pope N Laubach VE Su G. A novel role of IL-1β in NETosis and abdominal aortic aneurysms. Arterioscler Thromb Vasc Biol (2018) 38:843. doi: 10.1161/ATVBAHA.117.309897 29472233 PMC5864548

[47] Wei M Wang X Song Y Zhu D Qi D Jiao S. Inhibition of peptidyl arginine deiminase 4-dependent neutrophil extracellular trap formation reduces angiotensin II-induced abdominal aortic aneurysm rupture in mice. Front Cardiovasc Med (2021) 8:676612. doi: 10.3389/FCVM.2021.676612 34395553 PMC8360833

[48] Weisberg SP Hunter D Huber R Lemieux J Slaymaker S Vaddi K. CCR2 modulates inflammatory and metabolic effects of high-fat feeding. J Clin Invest (2006) 116:115–24. doi: 10.1172/JCI24335 PMC1307559 16341265

[49] Murphy AJ Akhtari M Tolani S Pagler T Bijl N Kuo CL. ApoE regulates hematopoietic stem cell proliferation, monocytosis, and monocyte accumulation in atherosclerotic lesions in mice. J Clin Invest (2011) 121:4138–49. doi: 10.1172/JCI57559 PMC3195472 21968112

[50] Croce K Gao H Wang Y Mooroka T Sakuma M Shi C. Myeloid-related protein-8/14 is critical for the biological response to vascular injury. Circulation (2009) 120:427–36. doi: 10.1161/CIRCULATIONAHA.108.814582 PMC3070397 19620505

[51] Combadiere C Potteaux S Rodero M Simon T Pezard A Esposito B. Combined inhibition of CCL2, CX3CR1, and CCR5 abrogates Ly6C hi and Ly6C lo monocytosis and almost abolishes atherosclerosis in hypercholesterolemic mice. Circulation (2008) 117:1649–57. doi: 10.1161/CIRCULATIONAHA.107.745091 18347211

[52] Christ A Günther P Lauterbach MAR Duewell P Biswas D Pelka K. Western Diet triggers NLRP3-dependent innate immune reprogramming. Cell (2018) 172:162–175.e14. doi: 10.1016/J.CELL.2017.12.013 29328911 PMC6324559

[53] Thatcher SE Zhang X Howatt DA Lu H Gurley SB Daugherty A. Angiotensin-converting enzyme 2 deficiency in whole body or bone marrow-derived cells increases atherosclerosis in low-density lipoprotein receptor-/- mice. Arterioscler Thromb Vasc Biol (2011) 31:758–65. doi: 10.1161/ATVBAHA.110.221614 PMC3086633 21252069

[54] Tabas I Lichtman AH. Monocyte-macrophages and T cells in atherosclerosis. Immunity (2017) 47:621–34. doi: 10.1016/j.immuni.2017.09.008 PMC5747297 29045897

[55] Flores-Gomez D Bekkering S Netea MG Riksen NP. Trained immunity in atherosclerotic cardiovascular disease. Arterioscler Thromb Vasc Biol (2021) 41:62–9. doi: 10.1161/ATVBAHA.120.314216 33147995

[56] Langenskiöld M Smidfelt K Nordanstig J Bergström G Tivesten A. Leukocyte subsets and abdominal aortic aneurysms detected by screening in men. J Intern Med (2020) 288:345–55. doi: 10.1111/JOIM.13040 32173961

[57] Korbecki J Bajdak-Rusinek K Kupnicka P Kapczuk P Simińska D Chlubek D. The role of CXCL16 in the pathogenesis of cancer and other diseases. Int J Mol Sci (2021) 22:3490. doi: 10.3390/IJMS22073490 33800554 PMC8036711

[58] Dragoljevic D Kraakman MJ Nagareddy PR Ngo D Shihata W Kammoun HL. Defective cholesterol metabolism in haematopoietic stem cells promotes monocyte-driven atherosclerosis in rheumatoid arthritis. Eur Hear J (2018) 39:2158–67. doi: 10.1093/eurheartj/ehy119 PMC6001889 29905812

[59] Hilgendorf I Swirski FK Robbins CS. Monocyte fate in atherosclerosis. Arter Thromb Vasc Biol (2015) 35:272–9. doi: 10.1161/ATVBAHA.114.303565 25538208

[60] Owens 3AP Passam FH Antoniak S Marshall SM McDaniel AL Rudel L. Monocyte tissue factor-dependent activation of coagulation in hypercholesterolemic mice and monkeys is inhibited by simvastatin. J Clin Invest (2012) 122:558–68. doi: 10.1172/JCI58969 PMC3266787 22214850

[61] Knappich C Spin JM Eckstein HH Tsao PS Maegdefessel L. Involvement of myeloid cells and noncoding RNA in abdominal aortic aneurysm disease. Antioxid Redox Signal (2020) 33:602–20. doi: 10.1089/ARS.2020.8035 PMC7455479 31989839

[62] Ghigliotti G Barisione C Garibaldi S Brunelli C Palmieri D Spinella G. CD16+ monocyte subsets are increased in Large abdominal aortic aneurysms and are differentially related with circulating and cell-associated biochemical and inflammatory biomarkers. Dis Markers (2013) 34:131. doi: 10.3233/DMA-120956 23348634 PMC3809748

[63] Nahrendorf M Keliher E Marinelli B Leuschner F Robbins CS Gerszten RE. Detection of macrophages in aortic aneurysms by nanoparticle positron emission tomography-computed tomography. Arter Thromb Vasc Biol (2011) 31:750–7. doi: 10.1161/ATVBAHA.110.221499 PMC3060293 21252070

[64] Yu H Moran CS Trollope AF Woodward L Kinobe R Rush CM. Angiopoietin-2 attenuates angiotensin II-induced aortic aneurysm and atherosclerosis in apolipoprotein e-deficient mice. Sci Rep (2016) 6:35190. doi: 10.1038/srep35190 27767064 PMC5073347

[65] Tieu BC Lee C Sun H LeJeune W Recinos A Ju X. An adventitial IL-6/MCP1 amplification loop accelerates macrophage-mediated vascular inflammation leading to aortic dissection in mice. J Clin Invest (2009) 119:3637–51. doi: 10.1172/JCI38308 PMC2786788 19920349

[66] Zhou M Wang X Shi Y Ding Y Li X Xie T. Deficiency of ITGAM attenuates experimental abdominal aortic aneurysm in mice. J Am Hear Assoc Cardiovasc Cerebrovasc Dis (2021) 10:19900. doi: 10.1161/JAHA.120.019900 PMC8174368 33749307

[67] Furusawa J Mizoguchi I Chiba Y Hisada M Kobayashi F Yoshida H. Promotion of expansion and differentiation of hematopoietic stem cells by interleukin-27 into myeloid progenitors to control infection in emergency myelopoiesis. PloS Pathog (2016) 12:e1005507. doi: 10.1371/journal.ppat.1005507 26991425 PMC4798290

[68] Swirski FK Libby P Aikawa E Alcaide P Luscinskas FW Weissleder R. Ly-6Chi monocytes dominate hypercholesterolemia-associated monocytosis and give rise to macrophages in atheromata. J Clin Invest (2007) 117:195–205. doi: 10.1172/JCI29950 17200719 PMC1716211

[69] Nahrendorf M Swirski FK. Innate immune cells in ischaemic heart disease: does myocardial infarction beget myocardial infarction? Eur Hear J (2016) 37:868–72. doi: 10.1093/eurheartj/ehv453 PMC4789592 26351395

[70] Dutta P Hoyer FF Sun Y Iwamoto Y Tricot B Weissleder R. E-selectin inhibition mitigates splenic HSC activation and myelopoiesis in hypercholesterolemic mice with myocardial infarction. Arter Thromb Vasc Biol (2016) 36:1802–8. doi: 10.1161/ATVBAHA.116.307519 PMC5001901 27470513

[71] Leuschner F Rauch PJ Ueno T Gorbatov R Marinelli B Lee WW. Rapid monocyte kinetics in acute myocardial infarction are sustained by extramedullary monocytopoiesis. J Exp Med (2012) 209:123–37. doi: 10.1084/jem.20111009 PMC3260875 22213805

[72] Cui M Cai Z Chu S Sun Z Wang X Hu L. Orphan nuclear receptor Nur77 inhibits angiotensin II-induced vascular remodeling *via* downregulation of β-catenin. Hypertens (Dallas Tex 1979) (2016) 67:153–62. doi: 10.1161/HYPERTENSIONAHA.115.06114 26597820

[73] Gschwandtner M Derler R Midwood KS. More than just attractive: How CCL2 influences myeloid cell behavior beyond chemotaxis. Front Immunol (2019) 10:2759/BIBTEX. doi: 10.3389/FIMMU.2019.02759/BIBTEX 31921102 PMC6923224

[74] Rao J Brown BN Weinbaum JS Ofstun EL Makaroun MS Humphrey JD. Distinct macrophage phenotype and collagen organization within the intraluminal thrombus of abdominal aortic aneurysm. J Vasc Surg (2015) 62:585–93. doi: 10.1016/J.JVS.2014.11.086 PMC4550501 26206580

[75] Yan H Hu Y Akk A Ye K Bacon J Pham CTN. Interleukin-12 and -23 blockade mitigates elastase-induced abdominal aortic aneurysm. Sci Rep (2019) 9 :10447. doi: 10.1038/S41598-019-46909-Y 31320700 PMC6639297

[76] Tabas I. Macrophage death and defective inflammation resolution in atherosclerosis. Nat Rev Immunol (2010) 10:36–46. doi: 10.1038/nri2675 19960040 PMC2854623

[77] Zernecke A Bernhagen J Weber C. Macrophage migration inhibitory factor in cardiovascular disease. Circulation (2008) 117:1594–602. doi: 10.1161/CIRCULATIONAHA.107.729125 18362243

[78] Bae YS Lee JH Choi SH Kim S Almazan F Witztum JL. Macrophages generate reactive oxygen species in response to minimally oxidized low-density lipoprotein: Toll-like receptor 4- and spleen tyrosine kinase-dependent activation of NADPH oxidase 2. Circ Res (2009) 104:210–8. doi: 10.1161/CIRCRESAHA.108.181040 PMC2720065 19096031

[79] Riboldi E Porta C Morlacchi S Viola A Mantovani A Sica A. Hypoxia-mediated regulation of macrophage functions in pathophysiology. Int Immunol (2013) 25:67–75. doi: 10.1093/intimm/dxs110 23179187

[80] Qin Z Bagley J Sukhova G Baur WE Park HJ Beasley D. Angiotensin II-induced TLR4 mediated abdominal aortic aneurysm in apolipoprotein e knockout mice is dependent on STAT3. J Mol Cell Cardiol (2015) 87:160–70. doi: 10.1016/j.yjmcc.2015.08.014 26299839

[81] Johnston WF Salmon M Pope NH Meher A Su G Stone ML. Inhibition of interleukin-1beta decreases aneurysm formation and progression in a novel model of thoracic aortic aneurysms. Circulation (2014) 130:S51–9. doi: 10.1161/CIRCULATIONAHA.113.006800 PMC5097450 25200056

[82] Batra R Suh MK Carson JS Dale MA Meisinger TM Fitzgerald M. IL-1β and TNF-α impact abdominal aortic aneurysm formation by differential effects on macrophage polarization. Arterioscler Thromb Vasc Biol (2018) 38:457. doi: 10.1161/ATVBAHA.117.310333 29217508 PMC7450719

[83] Shi J Guo J Li Z Xu B Miyata M. Importance of NLRP3 inflammasome in abdominal aortic aneurysms. J Atheroscler Thromb (2021) 28:454. doi: 10.5551/JAT.RV17048 33678767 PMC8193780

[84] Li H Xu H Liu S. Toll-like receptors 4 induces expression of matrix metalloproteinase-9 in human aortic smooth muscle cells. Mol Biol Rep (2011) 38:1419–23. doi: 10.1007/S11033-010-0246-4 20725790

[85] Li T Li X Liu X Yang J Ma C. The elevated expression of TLR4 and MMP9 in human abdominal aortic aneurysm tissues and its implication. BMC Cardiovasc Disord (2021) 21:378. doi: 10.1186/S12872-021-02193-1 34348653 PMC8336015

[86] Maguire EM Pearce SWA Xiao R Oo AY Xiao Q. Matrix metalloproteinase in abdominal aortic aneurysm and aortic dissection. Pharmaceuticals (2019) 12:118. doi: 10.3390/PH12030118 PMC6789891 31390798

[87] Longo GM Xiong W Greiner TC Zhao Y Fiotti N Baxter BT. Matrix metalloproteinases 2 and 9 work in concert to produce aortic aneurysms. J Clin Invest (2002) 110:625–32. doi: 10.1172/JCI15334 PMC151106 12208863

[88] Grivennikov SI Tumanov AV Liepinsh DJ Kruglov AA Marakusha BI Shakhov AN. Distinct and nonredundant *in vivo* functions of TNF produced by t cells and macrophages/neutrophils: Protective and deleterious effects. Immunity (2005) 22:93–104. doi: 10.1016/j.immuni.2004.11.016 15664162

[89] Xiong W MacTaggart J Knispel R Worth J Persidsky Y Baxter BT. Blocking TNF-α attenuates aneurysm formation in a murine model. J Immunol (2009) 183:2741–6. doi: 10.4049/JIMMUNOL.0803164 PMC4028114 19620291

[90] Xanthoulea S Thelen M Pöttgens C Gijbels MJJ Lutgens E de Winther MPJ. Absence of p55 TNF receptor reduces atherosclerosis, but has no major effect on angiotensin II induced aneurysms in LDL receptor deficient mice. PloS One (2009) 4:e6113. doi: 10.1371/JOURNAL.PONE.0006113 19582157 PMC2702081

[91] Ju X Ijaz T Sun H Ray S Lejeune W Lee C. Interleukin-6-signal transducer and activator of transcription-3 signaling mediates aortic dissections induced by angiotensin II *via* the T-helper lymphocyte 17-interleukin 17 axis in C57BL/6 mice. Arterioscler Thromb Vasc Biol (2013) 33:1612–21. doi: 10.1161/ATVBAHA.112.301049 PMC3818154 23685554

[92] Han Y Runge MS Brasier AR. Angiotensin II induces interleukin-6 transcription in vascular smooth muscle cells through pleiotropic activation of nuclear factor-kappa b transcription factors. Circ Res (1999) 84:695–703. doi: 10.1161/01.RES.84.6.695 10189357

[93] Peshkova IO Schaefer G Koltsova EK. Atherosclerosis and aortic aneurysm - is inflammation a common denominator? FEBS J (2016) 283:1636–52. doi: 10.1111/febs.13634 26700480

[94] Sharma N Hans CP. Interleukin 12p40 deficiency promotes abdominal aortic aneurysm by activating CCN2/MMP2 pathways. J Am Heart Assoc (2021) 10:1–33. doi: 10.1161/JAHA.120.017633 PMC7955443 33470127

[95] Hadi T Boytard L Silvestro M Alebrahim D Jacob S Feinstein J. Macrophage-derived netrin-1 promotes abdominal aortic aneurysm formation by activating MMP3 in vascular smooth muscle cells. Nat Commun (2018) 9:5022. doi: 10.1038/S41467-018-07495-1 30479344 PMC6258757

[96] Wang Y Jia L Xie Y Cai Z Liu Z Shen J. Involvement of macrophage-derived exosomes in abdominal aortic aneurysms development. Atherosclerosis (2019) 289:64–72. doi: 10.1016/J.ATHEROSCLEROSIS.2019.08.016 31479773

[97] Jadli AS Parasor A Gomes KP Shandilya R Patel VB. Exosomes in cardiovascular diseases: Pathological potential of nano-messenger. Front Cardiovasc Med (2021) 8:767488. doi: 10.3389/FCVM.2021.767488 34869682 PMC8632805

[98] Watanabe S Alexander M Misharin AV Budinger GRS. The role of macrophages in the resolution of inflammation. J Clin Invest (2019) 129:2619–28. doi: 10.1172/JCI124615 PMC6597225 31107246

[99] Moore JP Vinh A Tuck KL Sakkal S Krishnan SM Chan CT. M2 macrophage accumulation in the aortic wall during angiotensin II infusion in mice is associated with fibrosis, elastin loss, and elevated blood pressure. Am J Physiol Heart Circ Physiol (2015) 309:H906–17. doi: 10.1152/AJPHEART.00821.2014 26071547

[100] Zhu H Qu X Zhang C Yu Y. Interleukin-10 promotes proliferation of vascular smooth muscle cells by inhibiting inflammation in rabbit abdominal aortic aneurysm. Int J Clin Exp Pathol (2019) 12:1260–71. PMC6947044 31933940

[101] Adam M Kooreman NG Jagger A Wagenhäuser MU Mehrkens D Wang Y. Systemic upregulation of IL-10 using a non-immunogenic vector reduces growth and rate of dissecting abdominal aortic aneurysm. Arterioscler Thromb Vasc Biol (2018) 38:1796. doi: 10.1161/ATVBAHA.117.310672 29880489 PMC6652227

[102] Lareyre F Clement M Raffort J Pohlod S Patel M Esposito B. TGFbeta (Transforming growth factor-beta) blockade induces a human-like disease in a nondissecting mouse model of abdominal aortic aneurysm. Arter Thromb Vasc Biol (2017) 37:2171–81. doi: 10.1161/ATVBAHA.117.309999 28912363

[103] Angelov SN Hu JH Wei H Airhart N Shi M D’Ichek DA. TGF-β (Transforming growth factor-β) signaling protects the thoracic and abdominal aorta from angiotensin II-induced pathology by distinct mechanisms. Arterioscler Thromb Vasc Biol (2017) 37:2102–13. doi: 10.1161/ATVBAHA.117.309401 PMC5658248 28729364

[104] Davies LC Taylor PR. Tissue-resident macrophages: then and now. Immunology (2015) 144:541. doi: 10.1111/IMM.12451 25684236 PMC4368161

[105] Yuan Z Lu Y Wei J Wu J Yang J Cai Z. Abdominal aortic aneurysm: Roles of inflammatory cells. Front Immunol (2021) 11:609161/BIBTEX. doi: 10.3389/FIMMU.2020.609161/BIBTEX 33613530 PMC7886696

[106] Gao Y Qian N Xu J Wang Y. The roles of macrophages in heart regeneration and repair after injury. Front Cardiovasc Med (2021) 0:744615. doi: 10.3389/FCVM.2021.744615 PMC8575035 34760943

[107] Zhao G Lu H Chang Z Zhao Y Zhu T Chang L. Single-cell RNA sequencing reveals the cellular heterogeneity of aneurysmal infrarenal abdominal aorta. Cardiovasc Res (2021) 117:1402. doi: 10.1093/CVR/CVAA214 32678909 PMC8064434

[108] Gomez D Owens GK. Smooth muscle cell phenotypic switching in atherosclerosis. Cardiovasc Res (2012) 95:156–64. doi: 10.1093/CVR/CVS115 PMC3388816 22406749

[109] Haider P Kral-Pointner JB Mayer J Richter M Kaun C Brostjan C. Neutrophil extracellular trap degradation by differently polarized macrophage subsets. Arterioscler Thromb Vasc Biol (2020) 40:2265. doi: 10.1161/ATVBAHA.120.314883 32673525 PMC7447175

[110] Amin HZ Sasaki N Yamashita T Mizoguchi T Hayashi T Emoto T. CTLA-4 protects against angiotensin II-induced abdominal aortic aneurysm formation in mice. Sci Rep (2019) 9. doi: 10.1038/S41598-019-44523-6 PMC6542846 31147569

[111] Chistiakov DA Orekhov AN Sobenin IA Bobryshev YV. Plasmacytoid dendritic cells: development, functions, and role in atherosclerotic inflammation. Front Physiol (2014) 5:279. doi: 10.3389/FPHYS.2014.00279 25120492 PMC4110479

[112] Krishna SM Moran CS Jose RJ Lazzaroni S Huynh P Golledge J. Depletion of CD11c+ dendritic cells in apolipoprotein e-deficient mice limits angiotensin II-induced abdominal aortic aneurysm formation and growth. Clin Sci (Lond) (2019) 133:2203–15. doi: 10.1042/CS20190924 31696215

[113] Tsuruda T Kato J Hatakeyama K Kojima K Yano M Yano Y. Adventitial mast cells contribute to pathogenesis in the progression of abdominal aortic aneurysm. Circ Res (2008) 102:1368–77. doi: 10.1161/CIRCRESAHA.108.173682 18451339

[114] Tomimori Y Manno A Tanaka T Futamura-Takahashi J Muto T Nagahira K. ASB17061, a novel chymase inhibitor, prevented the development of angiotensin II-induced abdominal aortic aneurysm in apolipoprotein e-deficient mice. Eur J Pharmacol (2019) 856:72403. doi: 10.1016/J.EJPHAR.2019.05.032 31128093

[115] Liu CL Wang Y Liao M Wemmelund H Ren J Fernandes C. Allergic lung inflammation aggravates angiotensin II-induced abdominal aortic aneurysms in mice. Arter Thromb Vasc Biol (2016) 36:69–77. doi: 10.1161/ATVBAHA.115.305911 PMC4690809 26543094

[116] Li J Deng Z Zhang X Liu F Yang C Shi GP. Deficiency of IgE protects mice from experimental abdominal aortic aneurysms. FASEB J (2020) 34:3091. doi: 10.1096/FJ.201902095RR 31909541 PMC7018578

[117] Gao R Liu D Guo W Ge W Fan T Li B. Meprin-α (Mep1A) enhances TNF-α secretion by mast cells and aggravates abdominal aortic aneurysms. Br J Pharmacol (2020) 177:2872–85. doi: 10.1111/BPH.15019 PMC7236073 32072633

[118] Whitman SC Rateri DL Szilvassy SJ Yokoyama W Daugherty A. Depletion of natural killer cell function decreases atherosclerosis in low-density lipoprotein receptor null mice. Arterioscler Thromb Vasc Biol (2004) 24:1049–54. doi: 10.1161/01.ATV.0000124923.95545.2c 14988092

[119] Whitman SC Ramsamy TA. Participatory role of natural killer and natural killer T cells in atherosclerosis: lessons learned from *in vivo* mouse studies. Can J Physiol Pharmacol (2006) 84:67–75. doi: 10.1139/Y05-159 16845892

[120] Forester ND Cruickshank SM Scott DJA Carding SR. Increased natural killer cell activity in patients with an abdominal aortic aneurysm. Br J Surg (2006) 93:46–54. doi: 10.1002/BJS.5215 16315339

[121] Hinterseher I Schworer CM Lillvis JH Stahl E Erdman R Gatalica Z. Immunohistochemical analysis of the natural killer cell cytotoxicity pathway in human abdominal aortic aneurysms. Int J Mol Sci (2015) 16:11196–212. doi: 10.3390/IJMS160511196 PMC4463696 25993291

[122] Levoin N Jean M Legembre P. CD95 structure, aggregation and cell signaling. Front Cell Dev Biol (2020) 8:314/BIBTEX. doi: 10.3389/FCELL.2020.00314/BIBTEX 32432115 PMC7214685

[123] Liu Z Fitzgerald M Meisinger T Batra R Suh M Greene H. CD95-ligand contributes to abdominal aortic aneurysm progression by modulating inflammation. Cardiovasc Res (2019) 115:807–18. doi: 10.1093/CVR/CVY264 PMC6432056 30428004

[124] Newland SA Mohanta S Clément M Taleb S Walker JA Nus M. Type-2 innate lymphoid cells control the development of atherosclerosis in mice. Nat Commun (2017) 8:15781. doi: 10.1038/NCOMMS15781 28589929 PMC5467269

[125] Robinette ML Fuchs A Cortez VS Lee JS Wang Y Durum SK. Transcriptional programs define molecular characteristics of innate lymphoid cell classes and subsets. Nat Immunol (2015) 16:306–17. doi: 10.1038/ni.3094 PMC4372143 25621825

[126] Helfrich S Mindt BC Fritz JH Duerr CU. Group 2 innate lymphoid cells in respiratory allergic inflammation. Front Immunol (2019) 10:930. doi: 10.3389/FIMMU.2019.00930 31231357 PMC6566538

[127] Neill DR Wong SH Bellosi A Flynn RJ Daly M Langford TKA. Nuocytes represent a new innate effector leukocyte that mediates type-2 immunity. Nature (2010) 464:1367. doi: 10.1038/NATURE08900 20200518 PMC2862165

[128] Brestoff JR Kim BS Saenz SA Stine RR Monticelli LA Sonnenberg GF. Group 2 innate lymphoid cells promote beiging of adipose and limit obesity. Nature (2015) 519:242. doi: 10.1038/NATURE14115 25533952 PMC4447235

[129] Sui Q Zhang J Sun X Zhang C Han Q Tian Z. NK cells are the crucial antitumor mediators when STAT3-mediated immunosuppression is blocked in hepatocellular carcinoma. J Immunol (2014) 193:2016–23. doi: 10.4049/jimmunol.1302389 25015826

[130] Eberl G Colonna M Di Santo JP McKenzie AN. Innate lymphoid cells. innate lymphoid cells: A new paradigm in immunology. Science (80-) (2015) 348:6566. doi: 10.1126/science.aaa6566 PMC5658207 25999512

[131] Roberts LB Lord GM Howard JK. Heartbreakers or healers? innate lymphoid cells in cardiovascular disease and obesity. Front Immunol (2022) 0:903678. doi: 10.3389/FIMMU.2022.903678 PMC9130471 35634348

[132] Crosby CM Kronenberg M. Tissue-specific functions of invariant natural killer T cells. Nat Rev Immunol (2018) 18:559–74. doi: 10.1038/s41577-018-0034-2 PMC6343475 29967365

[133] Kritikou E van Duijn J Nahon JE van der Heijden T Bouwman M Groeneveldt C. Disruption of a CD1d-mediated interaction between mast cells and NKT cells aggravates atherosclerosis. Atherosclerosis (2019) 280:132–9. doi: 10.1016/J.ATHEROSCLEROSIS.2018.11.027 30513408

[134] Miao T Wang T Feng T Yuan D Guo Q Xiong F. Activated invariant natural killer T cells infiltrate aortic tissue as key participants in abdominal aortic aneurysm pathology. Immunology (2021) 164:792–802. doi: 10.1111/IMM.13401 34379797 PMC8561115

[135] Saito A Ishimori N Tokuhara S Homma T Nishikawa M Iwabuchi K. Activation of invariant natural killer T cells by α-galactosylceramide attenuates the development of angiotensin II-mediated abdominal aortic aneurysm in obese ob/ob mice. Front Cardiovasc Med (2021) 8:659418. doi: 10.3389/FCVM.2021.659418 34041282 PMC8141584

[136] Albany CJ Trevelin SC Giganti G Lombardi G Scottà C. Getting to the heart of the matter: The role of regulatory T-cells (Tregs) in cardiovascular disease (CVD) and atherosclerosis. Front Immunol (2019) 10:2795/BIBTEX. doi: 10.3389/FIMMU.2019.02795/BIBTEX 31849973 PMC6894511

[137] Téo FH De Oliveira RTD Villarejos L Mamoni RL Altemani A Menezes FH. Characterization of CD4+ T cell subsets in patients with abdominal aortic aneurysms. Mediators Inflammation (2018) 2018. doi: 10.1155/2018/6967310 PMC6327259 30686933

[138] Puchenkova OA Soldatov VO Belykh AE Bushueva OY Piavchenko GA Venediktov AA. Cytokines in abdominal aortic aneurysm: Master regulators with clinical application. biomark Insights (2022) 17. doi: 10.1177/11772719221095676 PMC9052234 35492378

[139] Li D Li J Liu H Zhai L Hu W Xia N. Pathogenic tconvs promote inflammatory macrophage polarization through GM-CSF and exacerbate abdominal aortic aneurysm formation. FASEB J (2022) 36:e22172. doi: 10.1096/FJ.202101576R 35133017 PMC9303938

[140] Rosner D Stoneman V Littlewood T McCarthy N Figg N Wang Y. Interferon-gamma induces fas trafficking and sensitization to apoptosis in vascular smooth muscle cells *via* a PI3K- and akt-dependent mechanism. Am J Pathol (2006) 168:2054–63. doi: 10.2353/ajpath.2006.050473 PMC1606618 16723718

[141] Laurat E Poirier B Tupin E Caligiuri G Hansson GK Bariety J. *In vivo* downregulation of T helper cell 1 immune responses reduces atherogenesis in apolipoprotein e-knockout mice. Circulation (2001) 104:197–202. doi: 10.1161/01.cir.104.2.197 11447086

[142] Erbel C Sato K Meyer FB Kopecky SL Frye RL Goronzy JJ. Functional profile of activated dendritic cells in unstable atherosclerotic plaque. Basic Res Cardiol (2007) 102:123–32. doi: 10.1007/s00395-006-0636-x 17136419

[143] Xiong W Zhao Y Prall A Greiner TC Baxter BT. Key roles of CD4+ T cells and IFN-γ in the development of abdominal aortic aneurysms in a murine model. J Immunol (2004) 172:2607–12. doi: 10.4049/JIMMUNOL.172.4.2607 14764734

[144] Wang Y Ait-Oufella H Herbin O Bonnin P Ramkhelawon B Taleb S. TGF-β activity protects against inflammatory aortic aneurysm progression and complications in angiotensin II–infused mice. J Clin Invest (2010) 120:422–32. doi: 10.1172/JCI38136 PMC2810071 20101093

[145] Mousset CM Hobo W Woestenenk R Preijers F Dolstra H van der Waart AB. Comprehensive phenotyping of T cells using flow cytometry. Cytometry A (2019) 95:647–54. doi: 10.1002/CYTO.A.23724 30714682

[146] King VL Cassis LA Daugherty A. Interleukin-4 does not influence development of hypercholesterolemia or angiotensin II-induced atherosclerotic lesions in mice. Am J Pathol (2007) 171:2040–7. doi: 10.2353/ajpath.2007.060857 PMC2111126 18055554

[147] Davenport P Tipping PG. The role of interleukin-4 and interleukin-12 in the progression of atherosclerosis in apolipoprotein e-deficient mice. Am J Pathol (2003) 163:1117–25. doi: 10.1016/S0002-9440(10)63471-2 PMC1868277 12937153

[148] Binder CJ Hartvigsen K Chang M-K Miller M Broide D Palinski W. IL-5 links adaptive and natural immunity specific for epitopes of oxidized LDL and protects from atherosclerosis. J Clin Invest (2004) 114:427–37. doi: 10.1172/JCI20479 PMC484976 15286809

[149] Xu J Ehrman B Graham LM Eagleton MJ. Interleukin-5 is a potential mediator of angiotensin II-induced aneurysm formation in apolipoprotein e knockout mice. J Surg Res (2012) 178:512–8. doi: 10.1016/j.jss.2011.12.016 PMC3394914 22459292

[150] Shimizu K Shichiri M Libby P Lee RT Mitchell RN. Th2-predominant inflammation and blockade of IFN-gamma signaling induce aneurysms in allografted aortas. J Clin Invest (2004) 114:300–8. doi: 10.1172/JCI19855 PMC449742 15254597

[151] Shimizu K Mitchell RN Libby P. Inflammation and cellular immune responses in abdominal aortic aneurysms. Arterioscler Thromb Vasc Biol (2006) 26:987–94. doi: 10.1161/01.ATV.0000214999.12921.4f 16497993

[152] Sagan A Mikolajczyk TP Mrowiecki W MacRitchie N Daly K Meldrum A. T Cells are dominant population in human abdominal aortic aneurysms and their infiltration in the perivascular tissue correlates with disease severity. Front Immunol (2019) 10:1979/BIBTEX. doi: 10.3389/FIMMU.2019.01979/BIBTEX 31552015 PMC6736986

[153] Chan WL Pejnovic N Hamilton H Liew TV Popadic D Poggi A. Atherosclerotic abdominal aortic aneurysm and the interaction between autologous human plaque-derived vascular smooth muscle cells, type 1 NKT, and helper T cells. Circ Res (2005) 96:675–83. doi: 10.1161/01.RES.0000160543.84254.F1 15731463

[154] Schönbeck U Sukhova GK Gerdes N Libby P. TH2 predominant immune responses prevail in human abdominal aortic aneurysm. Am J Pathol (2002) 161:499–506. doi: 10.1016/S0002-9440(10)64206-X 12163375 PMC1850720

[155] Ivanov II McKenzie BS Zhou L Tadokoro CE Lepelley A Lafaille JJ. The orphan nuclear receptor RORgammat directs the differentiation program of proinflammatory IL-17+ T helper cells. Cell (2006) 126:1121–33. doi: 10.1016/j.cell.2006.07.035 16990136

[156] Smith E Prasad KM Butcher M Dobrian A Kolls JK Ley K. Blockade of interleukin-17A results in reduced atherosclerosis in apolipoprotein e-deficient mice. Circulation (2010). doi: 10.1161/CIRCULATIONAHA.109.924886 PMC2929562 20368519

[157] Chen S Crother TR Arditi M. Emerging role of IL-17 in atherosclerosis. J Innate Immun (2010) 2:325–33. doi: 10.1159/000314626 PMC2895754 20505315

[158] Warnatsch A Ioannou M Wang Q Papayannopoulos V. Neutrophil extracellular traps license macrophages and Th17 cells for cytokine production in atherosclerosis. Science (2015) 349:316. doi: 10.1126/SCIENCE.AAA8064 26185250 PMC4854322

[159] Wei Z Wang Y Zhang K Liao Y Ye P Wu J. Inhibiting the Th17/IL-17A-Related inflammatory responses with digoxin confers protection against experimental abdominal aortic aneurysm. Arterioscler Thromb Vasc Biol (2014) 34:2429–38. doi: 10.1161/ATVBAHA.114.304435 25234817

[160] Taleb S Romain M Ramkhelawon B Uyttenhove C Pasterkamp G Herbin O. Loss of SOCS3 expression in T cells reveals a regulatory role for interleukin-17 in atherosclerosis. J Exp Med (2009) 206:2067–77. doi: 10.1084/jem.20090545 PMC2757872 19737863

[161] Liao M Liu C-L Lv B-J Zhang J-Y Cheng L Cheng X. Plasma cytokine levels and risks of abdominal aortic aneurysms: A population-based prospective cohort study. Ann Med (2015) 47:245–52. doi: 10.3109/07853890.2015.1019916 PMC4669056 25856542

[162] Vent-Schmidt J Han JM MacDonald KG Levings MK. The role of FOXP3 in regulating immune responses. Int Rev Immunol (2014) 33:110–28. doi: 10.3109/08830185.2013.811657 23947341

[163] Subramanian M Thorp E Hansson GK Tabas I. Treg-mediated suppression of atherosclerosis requires MYD88 signaling in DCs. J Clin Invest (2013) 123:179–88. doi: 10.1172/JCI64617 PMC3533292 23257360

[164] Xie JJ Wang J Tang TT Chen J Gao XL Yuan J. The Th17/Treg functional imbalance during atherogenesis in ApoE(-/-) mice. Cytokine (2009), 185–93. doi: 10.1016/j.cyto.2009.09.007. advanced. 19836260

[165] Barhoumi T Kasal DA Li MW Shbat L Laurant P Neves MF. T Regulatory lymphocytes prevent angiotensin II-induced hypertension and vascular injury. Hypertens (Dallas Tex 1979) (2011) 57:469–76. doi: 10.1161/HYPERTENSIONAHA.110.162941 21263125

[166] Yodoi K Yamashita T Sasaki N Kasahara K Emoto T Matsumoto T. Foxp3+ regulatory T cells play a protective role in angiotensin II-induced aortic aneurysm formation in mice. Hypertens (Dallas Tex 1979) (2015) 65:889–95. doi: 10.1161/HYPERTENSIONAHA.114.04934 25601931

[167] Camacho SA Heath WR Carbone FR Sarvetnick N LeBon A Karlsson L. A key role for ICAM-1 in generating effector cells mediating inflammatory responses. Nat Immunol (2001) 2:523–9. doi: 10.1038/88720 11376339

[168] Liu B Kong J An G Zhang K Qin W Meng X. Regulatory T cells protected against abdominal aortic aneurysm by suppression of the COX-2 expression. J Cell Mol Med (2019) 23:6766–74. doi: 10.1111/JCMM.14554 PMC6787467 31328426

[169] Chen Z Stockton J Mathis D Benoist C. Modeling CTLA4-linked autoimmunity with RNA interference in mice. Proc Natl Acad Sci U.S.A. (2006) 103:16400–5. doi: 10.1073/pnas.0607854103 PMC1637594 17060611

[170] Ali AJ Makings J Ley K. Regulatory T cell stability and plasticity in atherosclerosis. Cells (2020) 9:2665. doi: 10.3390/CELLS9122665 PMC7764358 33322482

[171] Butcher MJ Filipowicz AR Waseem TC McGary CM Crow KJ Magilnick N. Atherosclerosis-driven treg plasticity results in formation of a dysfunctional subset of plastic IFNγ+ Th1/Tregs. Circ Res (2016) 119:1190–203. doi: 10.1161/CIRCRESAHA.116.309764 PMC5242312 27635087

[172] Li J McArdle S Gholami A Kimura T Wolf D Gerhardt T. CCR5+T-bet+FoxP3+ effector CD4 T cells drive atherosclerosis. Circ Res (2016) 118:1540–52. doi: 10.1161/CIRCRESAHA.116.308648 PMC4867125 27021296

[173] Gaddis DE Padgett LE Wu R McSkimming C Romines V Taylor AM. Apolipoprotein AI prevents regulatory to follicular helper T cell switching during atherosclerosis. Nat Commun (2018) 9:1095. doi: 10.1038/S41467-018-03493-5 29545616 PMC5854619

[174] Meng L Lu Y Wang X Sui W Ge X Zhong M. Statin therapy protects against abdominal aortic aneurysms by inducing the accumulation of regulatory T cells in ApoE -/- mice. J Mol Med (Berl) (2022). doi: 10.1007/S00109-022-02213-3 35704059

[175] Kopacz A Werner E Grochot-Przeczek A Kloska D Hajduk K Neumayer C. Simvastatin attenuates abdominal aortic aneurysm formation favoured by lack of Nrf2 transcriptional activity. Oxid Med Cell Longev (2020). doi: 10.1155/2020/6340190 PMC7315306 32617140

[176] Kowalska K Habrowska-Górczynska DE Neumayer C Bolliger M Domenig C Piastowska-Ciesielska AW. Lower levels of caveolin-1 and higher levels of endothelial nitric oxide synthase are observed in abdominal aortic aneurysm patients treated with simvastatin. Acta Biochim Pol (2018) 65:111–8. doi: 10.18388/ABP.2017_2305 29549671

[177] Saigusa R Winkels H Ley K. T Cell subsets and functions in atherosclerosis. Nat Rev Cardiol (2020) 17:387. doi: 10.1038/S41569-020-0352-5 32203286 PMC7872210

[178] Baptista D Mach F Brandt KJ. Follicular regulatory T cell in atherosclerosis. J Leukoc Biol (2018) 104:925–30. doi: 10.1002/JLB.MR1117-469R 30134501

[179] Ghamar Talepoor A Khosropanah S Doroudchi M. Functional subsets of circulating follicular helper T cells in patients with atherosclerosis. Physiol Rep (2020) 8:e14637. doi: 10.14814/PHY2.14637 33230950 PMC7683878

[180] Roy P Orecchioni M Ley K. How the immune system shapes atherosclerosis: Roles of innate and adaptive immunity. Nat Rev Immunol (2022) 22:251–65. doi: 10.1038/S41577-021-00584-1 PMC10111155 34389841

[181] Burger F Miteva K Baptista D Roth A Fraga-Silva RA Martel C. Follicular regulatory helper T cells control the response of regulatory b cells to a high-cholesterol diet. Cardiovasc Res (2021) 117:743–55. doi: 10.1093/CVR/CVAA069 PMC7898950 32219371

[182] Duftner C Seiler R Klein-Weigel P Göbel H Goldberger C Ihling C. High prevalence of circulating CD4+CD28- T-cells in patients with small abdominal aortic aneurysms. Arterioscler Thromb Vasc Biol (2005) 25:1347–52. doi: 10.1161/01.ATV.0000167520.41436.c0 15845908

[183] Zhou H Yan H Cannon JL Springer LE Green JM Pham CTN. CD43-mediated IFN-γ production by CD8+ T cells promotes abdominal aortic aneurysm in mice. J Immunol (2013) 190:5078. doi: 10.4049/JIMMUNOL.1203228 23585675 PMC3647012

[184] Zhang S Kan X Li Y Li P Zhang C Li G. Deficiency of γδT cells protects against abdominal aortic aneurysms by regulating phosphoinositide 3-kinase/AKT signaling. J Vasc Surg (2018) 67:899–908.e1. doi: 10.1016/J.JVS.2016.03.474 28024851

[185] Seo IH Lee SJ Noh TW Kim JH Joo HC Shin EC. Increase of Vδ2+ T cells that robustly produce IL-17A in advanced abdominal aortic aneurysm tissues. Immune Netw (2021) 21:e17. doi: 10.4110/IN.2021.21.E17 33996173 PMC8099614

[186] Sage AP Tsiantoulas D Binder CJ Mallat Z. The role of b cells in atherosclerosis. Nat Rev Cardiol (2018) 16:180–96. doi: 10.1038/s41569-018-0106-9 30410107

[187] Meher AK Johnston WF Lu G Pope NH Bhamidipati CM Harmon DB. B2 cells suppress experimental abdominal aortic aneurysms. Am J Pathol (2014) 184:3130–41. doi: 10.1016/J.AJPATH.2014.07.006 PMC4215033 25194661

[188] Spinosa MD Montgomery WG Lempicki M Srikakulapu P Johnsrude MJ McNamara CA. B cell-activating factor antagonism attenuates the growth of experimental abdominal aortic aneurysm. Am J Pathol (2021) 191:2231–44. doi: 10.1016/J.AJPATH.2021.08.012 PMC8647430 34509440

[189] Schaheen B Downs EA Serbulea V Almenara CC Spinosa M Su G. B-cell depletion promotes aortic infiltration of immunosuppressive cells and is protective of experimental aortic aneurysm. Arter Thromb Vasc Biol (2016) 36:2191–202. doi: 10.1161/ATVBAHA.116.307559 PMC5083246 27634836

[190] Furusho A Aoki H Ohno-Urabe S Nishihara M Hirakata S Nishida N. Involvement of b cells, immunoglobulins, and syk in the pathogenesis of abdominal aortic aneurysm. J Am Hear Assoc Cardiovasc Cerebrovasc Dis (2018) 7:e007750. doi: 10.1161/JAHA.117.007750 PMC5907549 29545260

[191] Mikolajczyk TP Guzik TJ. Adaptive immunity in hypertension. Curr Hypertens Rep (2019) 21:68. doi: 10.1007/S11906-019-0971-6 31321561 PMC6647517

[192] Forrester SJ Booz GW Sigmund CD Coffman TM Kawai T Rizzo V. Angiotensin II signal transduction: An update on mechanisms of physiology and pathophysiology. Physiol Rev (2018) 98:1627. doi: 10.1152/PHYSREV.00038.2017 29873596 PMC6335102

[193] Sun P Zhang L Gu Y Wei S Wang Z Li M. Immune checkpoint programmed death-1 mediates abdominal aortic aneurysm and pseudoaneurysm progression. BioMed Pharmacother (2021) 142:111955. doi: 10.1016/J.BIOPHA.2021.111955 34339918

[194] Li Y Ren P Dawson A Vasquez HG Ageedi W Zhang C. Single-cell transcriptome analysis reveals dynamic cell populations and differential gene expression patterns in control and aneurysmal human aortic tissue. Circulation (2020) 142:1374–88. doi: 10.1161/CIRCULATIONAHA.120.046528 PMC7539140 33017217

[195] Wang X Zhang H Ge Y Cao L He Y Sun G. AT1R regulates macrophage polarization through YAP and regulates aortic dissection incidence. Front Physiol (2021) 12:644903/FULL. doi: 10.3389/FPHYS.2021.644903/FULL 34305627 PMC8299470

[196] Wang S Gu X Zhang Q Zhang X Li Y Yao Y. Angiotensin II suppresses rev-erbα expression in THP-1 macrophages *via* the ang II type 1 Receptor/Liver X receptor α pathway. Cell Physiol Biochem (2018) 46:303–13. doi: 10.1159/000488431 29590657

[197] Durik M Seva Pessoa B Roks AJ. The renin-angiotensin system, bone marrow and progenitor cells. Clin Sci (2012) 123:205–23. doi: 10.1042/CS20110660 22548406

[198] Peshkova IO Fatkhullina AR Mikulski Z Ley K Koltsova EK. IL-27R signaling controls myeloid cells accumulation and antigen-presentation in atherosclerosis. Sci Rep (2017) 7:2255. doi: 10.1038/s41598-017-01828-8 28536468 PMC5442117

[199] Swirski FK Nahrendorf M Etzrodt M Wildgruber M Cortez-Retamozo V Panizzi P. Identification of splenic reservoir monocytes and their deployment to inflammatory sites. Science (80-) (2009) 325:612–6. doi: 10.1126/science.1175202 PMC2803111 19644120

[200] Robbins CS Chudnovskiy A Rauch PJ Figueiredo JL Iwamoto Y Gorbatov R. Extramedullary hematopoiesis generates ly-6C(high) monocytes that infiltrate atherosclerotic lesions. Circulation (2012) 125:364–74. doi: 10.1161/CIRCULATIONAHA.111.061986 PMC3263762 22144566

[201] Cheema MU Pluznick JL. Gut microbiota plays a central role to modulate the plasma and fecal metabolomes in response to angiotensin II. Hypertension (2019) 74:184–93. doi: 10.1161/HYPERTENSIONAHA.119.13155 PMC6587218 31154901

[202] Tang WH Kitai T Hazen SL. Gut microbiota in cardiovascular health and disease. Circ Res (2017) 120:1183–96. doi: 10.1161/CIRCRESAHA.117.309715 PMC5390330 28360349

[203] Singh RK Chang HW Yan D Lee KM Ucmak D Wong K. Influence of diet on the gut microbiome and implications for human health. J Transl Med (2017) 15:73. doi: 10.1186/s12967-017-1175-y 28388917 PMC5385025

[204] Liu R Hong J Xu X Feng Q Zhang D Gu Y. Gut microbiome and serum metabolome alterations in obesity and after weight-loss intervention. Nat Med (2017) 23:859–68. doi: 10.1038/nm.4358 28628112

[205] Shen J Obin MS Zhao L. The gut microbiota, obesity and insulin resistance. Mol Asp Med (2013) 34:39–58. doi: 10.1016/j.mam.2012.11.001 23159341

[206] Pindjakova J Sartini C Lo R Rappa F Coupe B Lelouvier B. Gut dysbiosis and adaptive immune response in diet-induced obesity vs. systemic inflammation. Front Microbiol (2017) 8:1157. doi: 10.3389/FMICB.2017.01157 28690599 PMC5479914

[207] Karbach SH Schönfelder T Brandão I Wilms E Hörmann N Jäckel S. Gut microbiota promote angiotensin II–induced arterial hypertension and vascular dysfunction. J Am Hear Assoc Cardiovasc Cerebrovasc Dis (2016) 5:e003698. doi: 10.1161/JAHA.116.003698 PMC5079031 27577581

[208] Zhu W Gregory JC Org E Buffa JA Gupta N Wang Z. Gut microbial metabolite TMAO enhances platelet hyperreactivity and thrombosis risk. Cell (2016) 165:111–24. doi: 10.1016/j.cell.2016.02.011 PMC4862743 26972052

[209] Araujo JR Tazi A Burlen-Defranoux O Vichier-Guerre S Nigro G Licandro H. Fermentation products of commensal bacteria alter enterocyte lipid metabolism. Cell Host Microbe (2020) 27:358–375 e7. doi: 10.1016/j.chom.2020.01.028 32101704

[210] Marques FZ Nelson E Chu PY Horlock D Fiedler A Ziemann M. High-fiber diet and acetate supplementation change the gut microbiota and prevent the development of hypertension and heart failure in hypertensive mice. Circulation (2017) 135:964–77. doi: 10.1161/CIRCULATIONAHA.116.024545 27927713

[211] Koeth RA Wang Z Levison BS Buffa JA Org E Sheehy BT. Intestinal microbiota metabolism of l-carnitine, a nutrient in red meat, promotes atherosclerosis. Nat Med (2013) 19:576–85. doi: 10.1038/nm.3145 PMC3650111 23563705

[212] Jonsson AL Caesar R Akrami R Reinhardt C Hållenius FF Borén J. Impact of gut microbiota and diet on the development of atherosclerosis in apoe–/– mice. Arterioscler Thromb Vasc Biol (2018) 38:2318–26. doi: 10.1161/ATVBAHA.118.311233 PMC6166703 29903735

[213] Witkowski M Weeks TL Hazen SL. Gut microbiota and cardiovascular disease. Circ Res (2020) 127:553. doi: 10.1161/CIRCRESAHA.120.316242 32762536 PMC7416843

[214] Brown JM Hazen SL. Metaorganismal nutrient metabolism as a basis of cardiovascular disease. Curr Opin Lipidol (2014) 25:48–53. doi: 10.1097/MOL.0000000000000036 24362355 PMC4018574

[215] Cani PD Amar J Iglesias MA Poggi M Knauf C Bastelica D. Metabolic endotoxemia initiates obesity and insulin resistance. Diabetes (2007) 56:1761–72. doi: 10.2337/db06-1491 17456850

[216] Guevara-Cruz M Flores-Lopez AG Aguilar-Lopez M Sanchez-Tapia M Medina-Vera I Dıaz D. Improvement of lipoprotein profile and metabolic endotoxemia by a lifestyle intervention that modifies the gut microbiota in subjects with metabolic syndrome. J Am Heart Assoc (2019) 8:e012401. doi: 10.1161/JAHA.119.012401 31451009 PMC6755842

[217] Bartolomaeus H Balogh A Yakoub M Homann S Markó L Höges S. Short-chain fatty acid propionate protects from hypertensive cardiovascular damage. Circulation (2019) 139:1407. doi: 10.1161/CIRCULATIONAHA.118.036652 30586752 PMC6416008

[218] Wang Z Klipfell E Bennett BJ Koeth R Levison BS Dugar B. Gut flora metabolism of phosphatidylcholine promotes cardiovascular disease. Nature (2011) 472:57–63. doi: 10.1038/nature09922 21475195 PMC3086762

[219] Organ CL Otsuka H Bhushan S Wang Z Bradley J Trivedi R. Choline diet and its gut microbe-derived metabolite, trimethylamine n-oxide, exacerbate pressure overload-induced heart failure. Circ Heart Fail (2016) 9:e002314. doi: 10.1161/CIRCHEARTFAILURE.115.002314 26699388 PMC4943035

[220] Luo Y Chen GL Hannemann N Ipseiz N Kronke G Bauerle T. Microbiota from obese mice regulate hematopoietic stem cell differentiation by altering the bone niche. Cell Metab (2015) 22:886–94. doi: 10.1016/j.cmet.2015.08.020 26387866

[221] Davis FM Tsoi LC Melvin WJ denDekker A Wasikowski R Joshi AD. Inhibition of macrophage histone demethylase JMJD3 protects against abdominal aortic aneurysms. J Exp Med (2021) 218:e20201839. doi: 10.1084/JEM.20201839 33779682 PMC8008365

[222] Verhaar BJ Prodan A Nieuwdorp M Muller M. Gut microbiota in hypertension and atherosclerosis: A review. Nutrients (2020) 12:1–22. doi: 10.3390/NU12102982 PMC7601560 33003455

[223] Zhang K Yang S Huang Y Qin X Qu K Chen Y. Alterations in gut microbiota and physiological factors associated with abdominal aortic aneurysm. Med Nov Technol Devices (2022) 14:100122. doi: 10.1016/J.MEDNTD.2022.100122

[224] Xie J Lu W Zhong L Hu Y Li Q Ding R. Alterations in gut microbiota of abdominal aortic aneurysm mice. BMC Cardiovasc Disord (2020) 20:32. doi: 10.1186/S12872-020-01334-2 31992206 PMC6988222

[225] Edwards JM Roy S Tomcho JC Schreckenberger ZJ Chakraborty S Bearss NR. Microbiota are critical for vascular physiology: Germ-free status weakens contractility and induces sex-specific vascular remodeling in mice. Vascul Pharmacol (2020) 125–126:106633. doi: 10.1016/J.VPH.2019.106633 PMC7036036 31843471

[226] Miller YI Viriyakosol S Worrall DS Boullier A Butler S Witztum JL. Toll-like receptor 4-dependent and -independent cytokine secretion induced by minimally oxidized low-density lipoprotein in macrophages. Arterioscler Thromb Vasc Biol (2005) 25:1213–9. doi: 10.1161/01.ATV.0000159891.73193.31 15718493

[227] El Chartouni C Rehli M. Comprehensive analysis of TLR4-induced transcriptional responses in interleukin 4-primed mouse macrophages. Immunobiology (2010) 215:780–7. doi: 10.1016/j.imbio.2010.05.032 20692533

[228] Rotta G Edwards EW Sangaletti S Bennett C Ronzoni S Colombo MP. Lipopolysaccharide or whole bacteria block the conversion of inflammatory monocytes into dendritic cells *in vivo* . J Exp Med (2003) 198:1253–63. doi: 10.1084/jem.20030335jem.20030335 PMC2194237 14568983

[229] Fatkhullina AR Peshkova IO Dzutsev A Aghayev T McCulloch JA Thovarai V. An interleukin-23-Interleukin-22 axis regulates intestinal microbial homeostasis to protect from diet-induced atherosclerosis. Immunity (2018) 49:943–957 e9. doi: 10.1016/j.immuni.2018.09.011 30389414 PMC6257980

[230] Martinez FO Sica A Mantovani A Locati M. Macrophage activation and polarization. Front Biosci (2008) 13:453–61. doi: 10.2741/2692 17981560

[231] Fisher EA Feig JE Hewing B Hazen SL Smith JD. High-density lipoprotein function, dysfunction, and reverse cholesterol transport. Arterioscler Thromb Vasc Biol (2012) 32:2813–20. doi: 10.1161/ATVBAHA.112.300133 PMC3501261 23152494

[232] Meital LT Windsor MT Maynard AE Schulze K Magee R O’donnell J. Endotoxin tolerance in abdominal aortic aneurysm macrophages, *In vitro*: A case–control study. Antioxidants (2020) 9:896. doi: 10.3390/ANTIOX9090896 PMC7554856 32967278

[233] Fatkhullina AR Peshkova IO Koltsova EK. The role of cytokines in the development of atherosclerosis. Biochem (Mosc) (2016) 81:1358–70. doi: 10.1134/S0006297916110134 PMC5471837 27914461

[234] Restini CBA Fink GD Watts SW. Vascular reactivity stimulated by TMA and TMAO: Are perivascular adipose tissue and endothelium involved? Pharmacol Res (2021) 163:105273. doi: 10.1016/J.PHRS.2020.105273 33197599 PMC7855790

[235] Matsuzawa Y Nakahashi H Konishi M Sato R Kawashima C Kikuchi S. Microbiota-derived trimethylamine n-oxide predicts cardiovascular risk after STEMI. Sci Rep (2019) 9:1–11. doi: 10.1038/s41598-019-48246-6 31406181 PMC6690996

[236] Bennett BJ de Aguiar Vallim TQ Wang Z Shih DM Meng Y Gregory J. Trimethylamine-n-oxide, a metabolite associated with atherosclerosis, exhibits complex genetic and dietary regulation. Cell Metab (2013) 17:49–60. doi: 10.1016/j.cmet.2012.12.011 23312283 PMC3771112

[237] Koeth RA Levison BS Culley MK Buffa JA Wang Z Gregory JC. Gamma-butyrobetaine is a proatherogenic intermediate in gut microbial metabolism of l-carnitine to TMAO. Cell Metab (2014) 20:799–812. doi: 10.1016/j.cmet.2014.10.006 25440057 PMC4255476

[238] Hartiala J Bennett BJ Tang WH Wang Z Stewart AF Roberts R. Comparative genome-wide association studies in mice and humans for trimethylamine n-oxide, a proatherogenic metabolite of choline and l-carnitine. Arter Thromb Vasc Biol (2014) 34:1307–13. doi: 10.1161/ATVBAHA.114.303252 PMC4035110 24675659

[239] Li XS Obeid S Klingenberg R Gencer B Mach F Raber L. Gut microbiota-dependent trimethylamine n-oxide in acute coronary syndromes: a prognostic marker for incident cardiovascular events beyond traditional risk factors. Eur Hear J (2017) 38:814–24. doi: 10.1093/eurheartj/ehw582 PMC5837488 28077467

[240] Brown JM Hazen SL. Microbial modulation of cardiovascular disease. Nat Rev Microbiol (2018) 16:171–81. doi: 10.1038/nrmicro.2017.149 PMC5885760 29307889

[241] Hu J Xu J Shen S Zhang W Chen H Sun X. Trimethylamine n-oxide promotes abdominal aortic aneurysm formation by aggravating aortic smooth muscle cell senescence in mice. J Cardiovasc Transl Res (2022), 1–11. doi: 10.1007/S12265-022-10211-6/FIGURES/6 35143032 PMC9622512

[242] Kriaa A Bourgin M Potiron A Mkaouar H Jablaoui A Gérard P. Microbial impact on cholesterol and bile acid metabolism: Current status and future prospects. J Lipid Res (2019) 60:323–32. doi: 10.1194/JLR.R088989 PMC6358303 30487175

[243] Lau K Srivatsav V Rizwan A Nashed A Liu R Shen R. Bridging the gap between gut microbial dysbiosis and cardiovascular diseases. Nutrients (2017) 9:859. doi: 10.3390/NU9080859 PMC5579652 28796176

[244] Zhou W Cheng Y Zhu P Nasser MI Zhang X Zhao M. Implication of gut microbiota in cardiovascular diseases. Oxid Med Cell Longev (2020). doi: 10.1155/2020/5394096 PMC7533754 33062141

[245] Nemet I Saha PP Gupta N Zhu W Romano KA Skye SM. A cardiovascular disease-linked gut microbial metabolite acts *via* adrenergic receptors. Cell (2020) 180:862. doi: 10.1016/J.CELL.2020.02.016 32142679 PMC7402401

[246] Abu Bakar H Robert Dunn W Daly C Ralevic V. Sensory innervation of perivascular adipose tissue: A crucial role in artery vasodilatation and leptin release. Cardiovasc Res (2017) 113:962–72. doi: 10.1093/CVR/CVX062 28371926

[247] Ayala-Lopez N Thompson JM Watts SW. Perivascular adipose tissue’s impact on norepinephrine-induced contraction of mesenteric resistance arteries. Front Physiol (2017) 8:37. doi: 10.3389/fphys.2017.00037 28228728 PMC5296360

[248] Ye T Zhang G Liu H Shi J Qiu H Liu Y. Relationships between perivascular adipose tissue and abdominal aortic aneurysms. Front Endocrinol (Lausanne) (2021) 12:701. doi: 10.3389/FENDO.2021.704845/BIBTEX PMC8236981 34194399

[249] Sahar S Dwarakanath RS Reddy MA Lanting L Todorov I Natarajan R. Angiotensin II enhances interleukin-18 mediated inflammatory gene expression in vascular smooth muscle cells: A novel cross-talk in the pathogenesis of atherosclerosis. Circ Res (2005) 96:1064–71. doi: 10.1161/01.RES.0000168210.10358.f4 15860756

[250] Liu CL Ren J Wang Y Zhang X Sukhova GK Liao M. Adipocytes promote interleukin-18 binding to its receptors during abdominal aortic aneurysm formation in mice. Eur Heart J (2020) 41:2456. doi: 10.1093/EURHEARTJ/EHZ856 31821481 PMC8453281

[251] Liu R Nikolajczyk BS. Tissue immune cells fuel obesity-associated inflammation in adipose tissue and beyond. Front Immunol (2019) 10:1587. doi: 10.3389/FIMMU.2019.01587 31379820 PMC6653202

[252] Lumeng CN Deyoung SM Saltiel AR. Macrophages block insulin action in adipocytes by altering expression of signaling and glucose transport proteins. Am J Physiol Endocrinol Metab (2007) 292:E166–74. doi: 10.1152/ajpendo.00284.2006 PMC3888778 16926380

[253] Feuerer M Herrero L Cipolletta D Naaz A Wong J Nayer A. Lean, but not obese, fat is enriched for a unique population of regulatory T cells that affect metabolic parameters. Nat Med (2009) 15:930–9. doi: 10.1038/nm.2002 PMC3115752 19633656

[254] Police SB Thatcher SE Charnigo R Daugherty A Cassis LA. Obesity promotes inflammation in periaortic adipose tissue and angiotensin II-induced abdominal aortic aneurysm formation. Arterioscler Thromb Vasc Biol (2009) 29:1458–64. doi: 10.1161/ATVBAHA.109.192658 PMC2753598 19608970

[255] Sakaue T Suzuki J Hamaguchi M Suehiro C Tanino A Nagao T. Perivascular adipose tissue angiotensin II type 1 receptor promotes vascular inflammation and aneurysm formation. Hypertens (Dallas Tex 1979) (2017) 70:780–9. doi: 10.1161/HYPERTENSIONAHA.117.09512 28760942

[256] Szasz T Webb RC. Perivascular adipose tissue: More than just structural support. Clin Sci (2012) 122:1–12. doi: 10.1042/CS20110151 PMC3966487 21910690

[257] Aghamohammadzadeh R Unwin RD Greenstein AS Heagerty AM. Effects of obesity on perivascular adipose tissue vasorelaxant function: Nitric oxide, inflammation and elevated systemic blood pressure. J Vasc Res (2015) 52:299–305. doi: 10.1159/000443885 26910225 PMC4961268

[258] Saxton SN Withers SB Nyvad J Mazur A Matchkov V Heagerty AM. Perivascular adipose tissue contributes to the modulation of vascular tone *in vivo* . J Vasc Res (2019) 56:320–32. doi: 10.1159/000502689 31550717

[259] Ramirez JG O’Malley EJ Ho WSV. Pro-contractile effects of perivascular fat in health and disease. Br J Pharmacol (2017) 174:3482. doi: 10.1111/BPH.13767 28257140 PMC5610161

[260] Park SY Kim KH Seo KW Bae JU Kim YH Lee SJ. Resistin derived from diabetic perivascular adipose tissue up-regulates vascular expression of osteopontin *via* the AP-1 signalling pathway. J Pathol (2014) 232:87–97. doi: 10.1002/path.4286 24089355 PMC4285806

[261] Barp CG Bonaventura D Assreuy J. NO, ROS, RAS, and PVAT: More than a soup of letters. Front Physiol (2021) 12:640021/BIBTEX. doi: 10.3389/FPHYS.2021.640021/BIBTEX 33643076 PMC7902489

[262] Folkesson M Vorkapic E Gulbins E Japtok L Kleuser B Welander M. Inflammatory cells, ceramides, and expression of proteases in perivascular adipose tissue adjacent to human abdominal aortic aneurysms. J Vasc Surg (2017) 65:1171–1179.e1. doi: 10.1016/J.JVS.2015.12.056 26960947

[263] Srikakulapu P Upadhye A Rosenfeld SM Marshall MA McSkimming C Hickman AW. Perivascular adipose tissue harbors atheroprotective IgM-producing b cells. Front Physiol (2017) 8:719/BIBTEX. doi: 10.3389/FPHYS.2017.00719/BIBTEX 28970806 PMC5609437

